# A Universal, Highly Stable Dopant System for Organic Semiconductors Based on Lewis-Paired Dopant Complexes

**DOI:** 10.1021/acsenergylett.4c01278

**Published:** 2024-07-01

**Authors:** Osnat Zapata-Arteaga, Aleksandr Perevedentsev, Michela Prete, Stephan Busato, Paolo Sebastiano Floris, Jesika Asatryan, Riccardo Rurali, Jaime Martín, Mariano Campoy-Quiles

**Affiliations:** † Institut de Ciència de Materials de Barcelona, ICMAB-CSIC, Campus UAB, 08193 Bellaterra, Spain; ‡ Molecular Gate SL, calle Genova 11, 28004 Madrid, Spain; § Department of Materials, Eidgenössische Technische Hochschule (ETH) Zürich, Vladimir-Prelog-Weg 5, Zürich 8093, Switzerland; ∥ Universidade da Coruña, Campus Industrial de Ferrol, CITENI, Esteiro, 15403 Ferrol, Spain

## Abstract

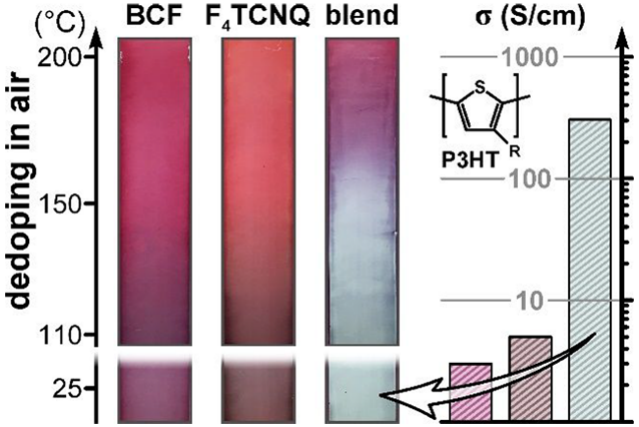

Chemical doping of organic semiconductors is an essential enabler for applications in electronic and energy-conversion devices such as thermoelectrics. Here, Lewis-paired complexes are advanced as high-performance dopants that address all the principal drawbacks of conventional dopants in terms of limited electrical conductivity, thermal stability, and generality. The study focuses on the Lewis acid B(C_6_F_5_)_3_ (BCF) and 2,3,5,6-tetrafluoro-7,7,8,8-tetracyanoquinodimethane (F_4_TCNQ) bearing Lewis-basic −CN groups. Due to its high electron affinity, BCF:F_4_TCNQ dopes an exceptionally wide range of organic semiconductors, over 20 of which are investigated. Complex activation and microstructure control lead to conductivities for poly(3-hexylthiophene) (P3HT) exceeding 300 and 900 S cm^–1^ for isotropic and chain-oriented films, respectively, resulting in a 10 to 50 times larger thermoelectric power factor compared to those obtained with neat dopants. Moreover, BCF:F_4_TCNQ-doped P3HT exhibits a 3-fold higher thermal dedoping activation energy compared to that obtained with neat dopants and at least a factor of 10 better operational stability.

The observation of doping-induced enhancement of electrical conductivity in halogen-exposed polyacetylene was, arguably, one of the pivotal reports that launched the molecular, “plastic” electronics field.^1^ Nearly five decades later it is evident that, in fact, nondoped organic semiconductors (OSCs) have witnessed the most rapid advance to application level in photovoltaics and photodetectors,^2−5^ while the successful use of doping in, e.g., charge-transport layers was the key stepping-stone toward widespread commercialization of organic light-emitting diodes.^6^ Clearly, molecular doping of OSCs represents a fundamental yet elusive enabler for the development of organic thermoelectrics with high power factors and field-effect transistors with low contact resistance, as well as improved photovoltaics, due to a number of challenging requirements.^7−9^ These include (i) maximal and homogeneous modulation of electrical conductivity upon doping, (ii) long-term stability of electrical characteristics under applied thermal and/or bias stress, (iii) suitability for high-throughput fabrication using solution-based methods, (iv) capability of spatial patterning of the doping level, and (v) preferably general applicability of the dopant to a broad range of OSCs.

The above-mentioned aspects are best illustrated by the example of molecular doping of benchmark semiconducting polymers. Typically, p-doping with molecular acceptors such as F_4_TCNQ relies on charge transfer (CT) from the highest occupied molecular orbital (HOMO) of the OSC to the lower-lying lowest unoccupied molecular orbital (LUMO) of the dopant, with both integer CT and CT complex pathways identified and studied in detail.^10,11^ Vapor-phase doping of P3HT^12^—a model polymer in the field—with F_4_TCNQ yields conductivities reaching 48 S cm^–1^, while the more scalable solution-based approaches typically result in lower values of 0.3 and 5.5 S cm^–1^ for co- and sequentially processed films, respectively.^13,14^ However, F_4_TCNQ cannot be used to dope high-mobility materials such as C_16_–IDTBT,^15^ PCDTBT^16^ and numerous others, which feature HOMO levels lying deeper than the LUMO of F_4_TCNQ (*E*_LUMO_ = −5.2 eV). Simultaneously, its relatively small size and reactivity of its specific functional groups^13^ lead to the rapid deterioration of the electrical and thermoelectric characteristics of F_4_TCNQ-doped materials at moderate temperatures of 100 °C^11,17^ or under applied bias in contact-doped organic field-effect transistors (OFETs).^8^ These and other factors stimulate extensive research into alternative CT dopants, such as Magic blue,^16^ molybdenum dithiolene complexes,^18^ F_6_TCNNQ^19^ and others.^9^ However, the increased synthetic complexity compromises the cost-efficiency underpinning the application potential of organic electronic materials. As an illustration, replacing F_4_TCNQ with F_6_TCNNQ gains an increase of *E*_LUMO_ by 0.13 eV and molar mass increase by a factor of 1.3 at a 4-fold increase in material cost from a typical supplier.^20^

Lewis acids are an alternative class of p-dopants,^21^ with Iron(III) chloride (FeCl_3_) being the most common, albeit unstable, example, and tris(pentafluorophenyl)borane (BCF) representing a more promising dopant due to its excellent solubility, higher stability and low cost. The specific p-doping mechanism by BCF, although still debated in the field,^22,23^ is most likely not a direct CT process to the LUMO of BCF but rather relies on the intermediate formation of a BCF:H_2_O complex exhibiting strong Bro̷nsted acidity.^24^ This ambiguity is exemplified by the broad distribution of experimental and calculated *E*_LUMO_ values reported for BCF that range from −3.0 to −5.3 eV.^25−28^ Nevertheless, BCF has been used to dope a variety of OSCs, generally enabling conductivities that are only marginally lower than those obtained for solution-based doping with F_4_TCNQ, e.g., 10 S cm^–1^ for P3HT.^29^

The recently emerged two-component dopant systems appear to offer a promising way forward. Ion-exchange doping involves conventional molecular doping with, e.g., F_4_TCNQ or FeCl_3_ as the first step, followed by dopant anion exchange with, e.g., TFSI^–^ upon exposure to its ionic solution.^30^ While this typically enables higher conductivities (up to 200 S cm^–1^ for P3HT^30^), the stability of the improved electrical characteristics is found to be relatively poor. Variants of the ion-exchange approach such as “cascade doping”^31^ furthermore appear to have limited applicability for many of the benchmark OSCs. Lewis acid–base pair dopants are also increasingly studied due to their ease of processing and wide parameter space offered by material selection. BCF paired with benzoyl peroxide—a weak Lewis base—was shown to efficiently dope P3HT despite significant microstructural disruption at large counterion concentrations.^32^ More recently, blends of BCF and −CN-bearing molecules such as F_4_TCNQ were shown to form complexes with strong oxidizing properties^33^ that may dope various OSCs.^28,34^ This was attributed to Lewis pairing of the boron center of BCF with the nitrile groups, leading to a 2-fold increase of the latter’s electron-withdrawing properties. Hence, the resulting BCF:F_4_TCNQ complexes featured *E*_LUMO_ of −5.9 eV, enabling p-doping of a wide range of OSCs, although the maximum obtained conductivities on the order of 10–20 S cm^–1^ did not reflect the full potential of this system.

Below we report the remarkable synergistic advantages of Lewis-paired dopants. In particular, we present a detailed study into the thermally induced stoichiometric BCF:F_4_TCNQ complex formation and fine-tuned doping characteristics with the aim of obtaining a solution-based doping method performed entirely under ambient conditions that enable state-of-the-art electrical conductivities in a roll-to-roll- (R2R-) compatible process. The results are benchmarked against neat, nonblended dopants throughout, emphasizing the outstanding ability of BCF:F_4_TCNQ to efficiently p-dope a wide range of materials with *E*_LUMO_ as low as −5.9 eV. Finally, Arrhenius analysis of thermal stability, as well as the demonstration of further charge-transport characteristics enhancement via microstructure engineering, confirm the prospect of Lewis-paired complexes for enabling a step-change in the performance and application potential of doped organic semiconductors.

P3HT is selected as the model semiconducting polymer given its ability to be doped by both F_4_TCNQ and BCF due to favorable energy level alignment (Figure 1a,b). Solution-sequential processing was adopted, whereby the dopant solutions were blade- or spin-coated directly onto P3HT films from solutions in acetonitrile:ethyl acetate–a nonsolvent mixture for typical OSCs that nevertheless allow for sufficient wetting and the formation of a macroscopically coherent dopant overlayer. The obtained P3HT:dopant (quasi-)bilayer films were then briefly annealed in air for 10 s at 90–120 °C to enable doping via (i) dopant diffusion into the OSC layer and, in the case of BCF:F_4_TCNQ blends, (ii) simultaneously activating dopant complexation (vide infra). UV-vis-NIR absorption spectra (Figure 1c) demonstrate the occurrence of doping with both BCF and F_4_TCNQ by the emergence of the characteristic P1 polaron feature (HOMO of P3HT → lower polaron band transition) above 1800 nm and partial bleaching of neutral polymer absorption at 511 nm. Absorption of ionised dopants partially overlaps with the P2 polaron feature (polaron interband transition) centered at 800 nm.

**Figure 1:**
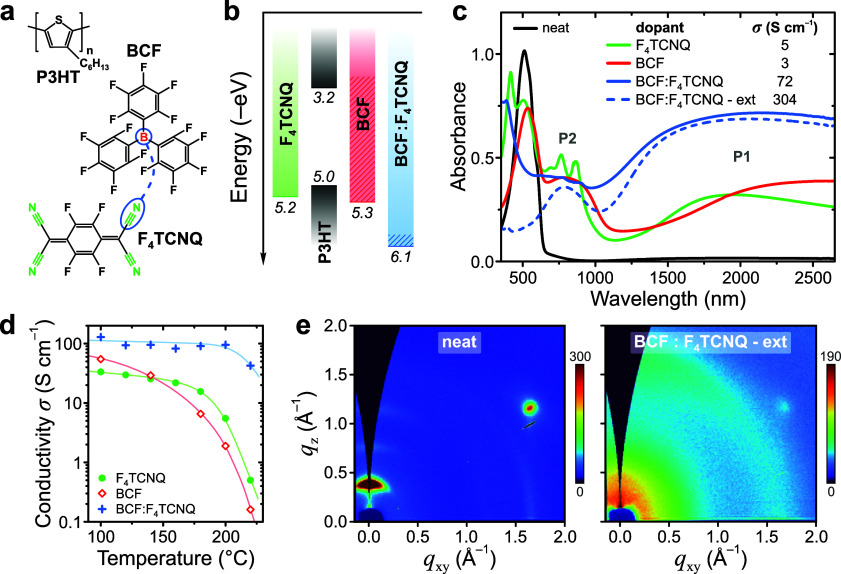
Illustration of P3HT doping with neat and Lewis-paired dopants. (**a**) Chemical structures of the principal materials used in this work and (**b**) the corresponding HOMO–LUMO levels of P3HT and LUMO levels of the dopants. For BCF and BCF:F_4_TCNQ complex, the deepest-lying LUMO levels are given while the shaded areas indicate the spread of values reported in the literature. (**c**) Absorption spectra and electrical conductivity (σ) values for P3HT films doped with the three dopant systems. In the case of BCF:F4TCNQ, data is shown for both the as-doped film and the same film following solvent-based extraction (“-ext”). (**d**) Electrical conductivity of doped P3HT films after sequential annealing under N_2_ atmosphere for 10 s at increasing temperatures. (**e**) 2D GIWAXS patterns for neat- and BCF:F_4_TNQ-doped P3HT films.

In the case of doping using a 10:1 wt/wt BCF:F_4_TCNQ blend (∼5:1 mol/mol; hereafter referred to as “blend-doping”), the optical signatures of doping become much stronger, with a more pronounced bleaching of neutral polymer absorption and higher amplitude of the P1 band. These observations are consistent with the respective electrical conductivity values (inset of Figure 1c) which increase by an order of magnitude for BCF:F_4_TCNQ blend-doped P3HT films in comparison to the individual dopants, reaching 72 S cm^–1^. Interestingly, the conductivity of blend-doped films can be further enhanced 4-fold to reach >300 S cm^–1^ via solvent-based extraction of excess dopant using “spin-off” with acetonitrile as the optimal dopant solvent. While this will be examined in detail in the following sections, here we note that this postprocessing step eliminates neutral dopant absorption at ∼400 nm and reveals a near-complete bleaching of the neutral polymer absorption band (Figure 1c), consistent with a very high doping level. Finally, to support subsequent analysis of thermal dedoping using absorption spectroscopy, we confirm an approximately linear relation between conductivity and the respective polaron to neutral band absorption ratios for these films (Supporting Information, Figure S1).

Further differences between individual- and blend-doped P3HT films are revealed by examining the doping stability and thin-film microstructure. Progressive annealing at increasing temperatures leads to rapid loss of conductivity for BCF- and F_4_CNQ-doped films above 150 °C (Figure 1d), while the conductivity of blend-doped films remains comparatively stable and more than 2 orders of magnitude higher following heating to 220 °C. GIWAXS data (Figure 1e) for neat P3HT reveals the usual semicrystalline microstructure with edge-on orientation of polymer chains. By comparison, the BCF:F_4_TCNQ-doped film is essentially amorphous. These observations are in stark contrast to the typical microstructure of ion-exchange-doped P3HT films which, although reaching comparably high conductivities,^30^ retain the high degree of microstructural order and exhibit only relatively minor changes in the lamellar and π–π spacings. (See Figures S2–S5, for reference GIWAXS data and full analysis details.) Taken together, these observations provide the first indication for the complexation of BCF:F_4_TCNQ, resulting in a substantially larger dopant molecule which exhibits higher thermal stability and introduces a considerable change in the microstructure of the host P3HT films.

Complexation between BCF and F_4_TCNQ is examined for solution-processed films and solvent-free powder blends. Figure 2a shows that the absorption spectra of as-spin-coated BCF:F_4_TCNQ blend films at varying molar compositions are, in essence, a convolution of the respective spectra for neat dopants. Following brief annealing at 90 °C, as performed for the above-described doping process, a vibronic progression appears for blend films above 700 nm which resembles absorption of the F_4_TCNQ^–^ anion,^35^ albeit with a 55 nm redshift. Absorption intensity at 910 nm relative to maximum absorption at ∼400 nm increases sharply above 2:1 mol/mol and reaches a maximum at 10:1 mol/mol BCF:F_4_TCNQ (Figure S6). Simultaneously, in the IR region annealing results in attenuation of the *υ*(C≡N) mode of neutral F_4_TCNQ at 2227 cm^–1^ and emergence of an intense peak at 2296 cm^–1^ which previous reports^28^ assign to the B···N stretching mode (Figure 2b, with reference DFT-calculated IR spectra shown in Figure S7). Raman spectroscopy can be used to selectively probe the distinct absorption peaks observed for annealed BCF:F_4_TCNQ blends above 700 nm by comparison of spectra recorded with resonant (785 nm) and nonresonant (488 nm) excitation. Resonant excitation reveals an additional pair of low-frequency modes at 401 and 429 cm^–1^ alongside the peak pair at 300 and 346 cm^–1^ seen for nonresonant excitation of neat- and blended F_4_TCNQ (Figures S8–S9). Vibrational modes in this region can be assigned to R−C≡N bending modes^36^ modified as a result of Lewis pairing with BCF in the annealed blend films. Finally, optical microscopy of annealed films (Figure S10) shows that while the neat dopants form crystalline films, vitrification occurs for blend films above 1:1 mol/mol BCF:F_4_TCNQ blending ratio.

**Figure 2:**
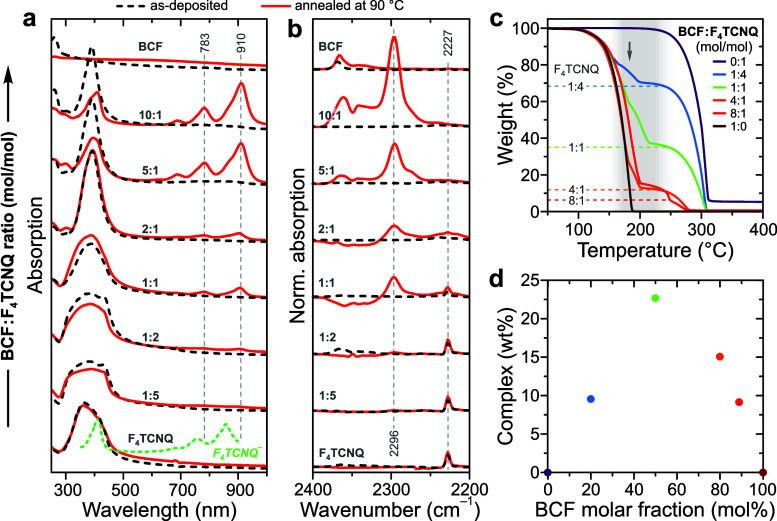
BCF:F_4_TCNQ Lewis-paired complex formation. (**a**) UV-vis-NIR (not normalized) and (**b**) FTIR spectra (normalized by the C=C peak at 960 cm^–1^) of BCF:F_4_TCNQ blend films at varying molar compositions. Data is shown for as-spin-coated and thermally annealed films. Also shown in (a) is the F_4_TCNQ^–^ anion absorption spectrum from ref (35). (**c**) TGA thermograms of dry-mixed BCF:F_4_TCNQ blends at varying molar compositions. Dotted lines indicate the nominal F_4_TCNQ wt % fraction in each sample. The shaded region highlights the distinct features of the complex observed for BCF:F_4_TCNQ blends. (**d**) Mass fraction of BCF:F_4_TCNQ complex estimated from the wt % span of the plateau-like regions in the 165–250 °C range.

BCF:F_4_TCNQ complexation is studied further by thermogravimetric analysis (TGA) and differential scanning calorimetry (DSC) for dry-mixed powder blends. TGA traces for neat dopants show monotonic weight loss with onset at 115 and 240 °C for BCF and F_4_TCNQ, respectively (Figure 2c). However, the data for BCF:F_4_TCNQ blends reveals the presence of an additional metastable species, for which maximum weight loss occurs at intermediate temperatures (Figure 2c, shaded region). DSC thermograms for BCF:F_4_TCNQ blends display the signature of an irreversible monotropic solid–solid transition in the 75–110 °C range of only the first-heating traces (Figures S11–S12), which is unambiguously assigned to complex formation. This shows the importance of the annealing step in “activating” the doping capability of the blend. Taken together, the stoichiometry of the complex can be estimated from the weight loss fraction that is spanned by the distinct plateau-like regions of the TGA traces for BCF:F_4_TCNQ blends. Figure 2d shows that the total estimated fraction of the complex peaks at 23 wt % for the 1:1 mol/mol blend, suggesting a 1:1 BCF:F_4_TCNQ complex stoichiometry. It should be noted, however, that this simple analysis can provide only an underestimated stoichiometry value in comparison to solution-deposited films given that the long-range molecular mobility is severely restricted for low-density powder blends.

The formation of the BCF:F_4_TCNQ complex is further supported by density-functional theory (DFT) calculations. We tested several possible binding geometries, finding that the most stable configuration features a bond formed between the B atom of BCF and one of the N atoms of F_4_TCNQ (Figure S13). The B···N distance within the complex (1.59 Å) is substantially smaller than the sum of the corresponding van der Waals radii (1.82 Å (B) + 1.54 Å (N) = 3.36 Å).^37^ The binding energy, computed as the difference between the total energy of the BCF:F_4_TCNQ complex and the isolated BCF and F_4_TCNQ molecules is −0.24 eV. We found another marginally stable complex geometry but, due to its low binding energy of only −11 meV, it is unlikely to be observed at room temperature.

Overall, the spectroscopic, thermal and DFT analyses support the occurrence of BCF:F_4_TCNQ complex formation. Complexation is thermally activated by heating above 75 °C without requiring a specific organic solvent and proceeds by Lewis pairing of the −C≡N groups of F_4_TCNQ with the boron center of BCF, as evidenced by vibrational spectroscopy and DFT calculations. The complexes thus contain an ionised F_4_TCNQ core coordinated with up to four BCF molecules, which is fully consistent with the very recent model proposed by Suh et al.^28^ The actual complex stoichiometry within doped films can plausibly fall short of 4:1 mol/mol depending on specific thin-film processing and kinetics thereof. Nevertheless, we note that P3HT films doped with BCF:F_4_TCNQ typically exhibit maximal electrical conductivities and thermoelectric power factors for blending ratios between 2:1 and 10:1 mol/mol (Figures S14–S15).

While the first indication of higher thermal stability of blend-doped P3HT under N_2_ atmosphere was provided in Figure 1d and other work,^28^ here it is re-examined for prolonged thermal exposure in air. Figure 3a,b shows transmitted-light images of large-area P3HT films before and after thermal dedoping on a Kofler bench spanning 110–200 °C across the substrate length. The distinct visual appearance of the pristine blend-doped film arises due to near-complete bleaching of neutral P3HT absorption and a contribution from the P2 band centered at 807 nm. The thermal stability of different dopants is analyzed using absorption spectra recorded at sample locations corresponding to specific annealing temperatures (Figure 3c). Comparison of film images and the corresponding absorption spectra highlight rapid dedoping for neat BCF and F_4_TCNQ dopants by 140–150 °C. The blend-doped film, however, retains clear signatures of its P1 and P2 polaron bands up to 200 °C. Generally, thermal dedoping of OSCs can proceed by three principal routes, namely: (i) physical loss of dopants by sublimation, (ii) disappearance of CT states and (iii) reaction of dopant molecules that yields effectively weaker dopants.^13^ In the case of doping by Lewis-paired complexes, dedoping is also possible by complex dissociation at elevated temperatures. Examination of the Raman spectra recorded across the range of annealing temperatures (Figure S16) highlights their continuous evolution between the reference spectra of as-doped and neat (nondoped) P3HT, thereby ruling out BCF:F_4_TCNQ dissociation or side-reaction with P3HT which would yield discontinuous or irreversible spectral changes, respectively. Hence, thermal dedoping of blend-doped P3HT is proposed to occur primarily by physical loss of dopant with onset at ∼150 °C (cf. TGA data in Figure 2c).

**Figure 3:**
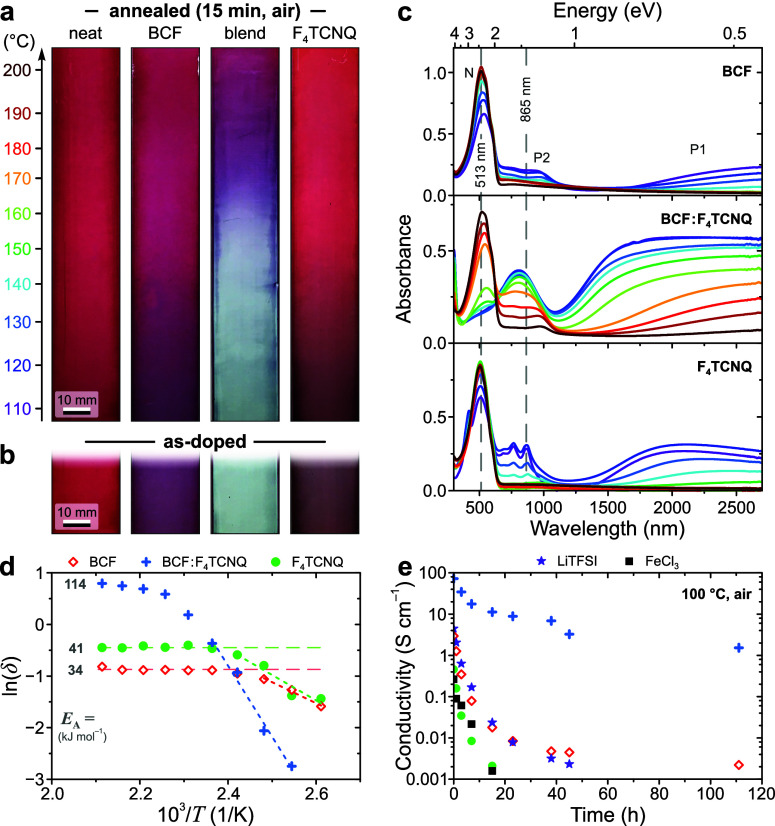
Thermal dedoping of P3HT films doped with BCF, F_4_TCNQ and 10:1 mol/mol BCF:F_4_TCNQ blend. (**a**) Transmitted-light images of doped P3HT films following thermal annealing along a temperature gradient spanning 110–200 °C across the substrate for 15 min in air and (**b**) sections of the same films prior to annealing. (**c**) Absorption spectra for the doped films shown in (a) as a function of annealing temperature. (**d**) Arrhenius plots for dedoping δ as a function of temperature *T* and the corresponding activation energies *E*_A_ obtained from linear fits (dotted lines). (**e**) “Operational” stability of doped P3HT films, including data for FeCl_3_- and LiTFSI-doped samples, showing electrical conductivity as a function of prolonged thermal annealing at 100 °C in air.

We employ Arrhenius analysis to quantitatively compare the thermal dedoping process for the three studied dopants. The degree of dedoping δ is defined as the difference in room-temperature electrical conductivities for as-doped and annealed films (σ_0_–σ). The conductivity of doped P3HT was shown by previous reports^13^ and Figure S1, to be closely approximated over a wide range of values by the intensity ratio of P2 (865 nm, 1.43 eV) and neutral polymer (513 nm, 2.42 eV) absorption, that is: P2/N. Hence, dedoping is expressed as exp(−*E*_A_/*RT*), where *E*_A_ is the activation energy and *R* is the gas constant, with the corresponding Arrhenius plots for the three dopants shown in Figure 3d. The *E*_A_ values obtained from linear fits are summarized in Table 1. While the dedoping activation energies are comparable for BCF- and F_4_TCNQ-doped P3HT, blend-doped P3HT exhibits a 3-fold higher *E*_A_, consistent with the larger volume of the BCF:F_4_TCNQ complex.

**Table 1:** Activation Energies *E*_A_ for Thermal Dedoping of P3HT Calculated from Absorption Ratios at 865 and 513 nm as a Function of Annealing Temperature, and Dopant Volumes Estimated from the Sum of van der Waals Radii

**Dopant**	**Dedoping *E*_**A**_(kJ mol^–1^)**	**Volume (Å^3^)**
BCF:F_4_TCNQ	114 ± 10	927^a^
BCF	34 ± 4	572
F_4_TCNQ	41 ± 9	355

a Value for 1:1 BCF:F_4_TCNQ complex.

To complete the comparison of relative thermal stabilities for different dopants, “operational” stability was measured for doped P3HT under prolonged (>100 h) thermal annealing at 100 °C in air, including reference films doped with BCF, F_4_TCNQ, FeCl_3_ and ion-exchange doped with bis(trifluoromethane)sulfonimide lithium salt (LiTFSI). Such protocol is comparable to the operating conditions for an OSC-based thermoelectric device or an accelerated-aging test for a contact-doped OFET. Clearly, BCF-F_4_TCNQ-doped P3HT exhibits superior thermal stability compared with all other dopants (Figure 3e; extended-range data in Figure S17). In particular, while P3HT doped with BCF-F_4_TCNQ exhibits ×20 higher conductivity than LiTFSI for pristine films, the ratio reaches ×100 after 7 h and exceeds ×1000 after 20 h. Alternatively, we can quantify the stability by the rate of conductivity loss, which is at least 10 times slower for the complex compared to any of the neat dopants.

Lewis-paired BCF:F_4_TCNQ complex features a deep LUMO level of −5.9 eV measured experimentally,^28^ in fair agreement with our theoretical estimate of −6.1 eV (*N.B*. according to ref (28) the agreement further improves by using a B3LYP functional for the exchange-correlation energy). Hence, BCF:F_4_TCNQ was shown to p-dope a wide range of OSCs, including n-type materials such as N2200 with *E*_HOMO_ = −5.8 eV. However, electrical conductivities reached only 10–20 S cm^–1^, while the values for OSCs with deep-lying HOMO levels, such as N2200, were not reported.^28^ Here we apply a consistent doping protocol, comprising blade-coating of the dopant solutions followed by brief thermal “activation” (see Materials and methods; Supporting Information), to dope a range of materials using BCF, F_4_TCNQ and 10:1 wt/wt BCF:F_4_TCNQ. The results for 20 small-molecular and polymeric OSCs are summarized in Figure 4a, with additional data presented in Figure 4b and Figures S18–S19. In all cases, data is shown for the as-doped films (that is, without any additional solvent-extraction steps) which, by analogy with P3HT (Figure 1c), implies that still higher electrical conductivities may be reached by further optimization. Nevertheless, sample images in Figure 4b highlight that homogeneous doping over large ∼8 × 8 mm^2^ film areas is generally achieved with the adopted roll-to-roll-compatible processing.

**Figure 4:**
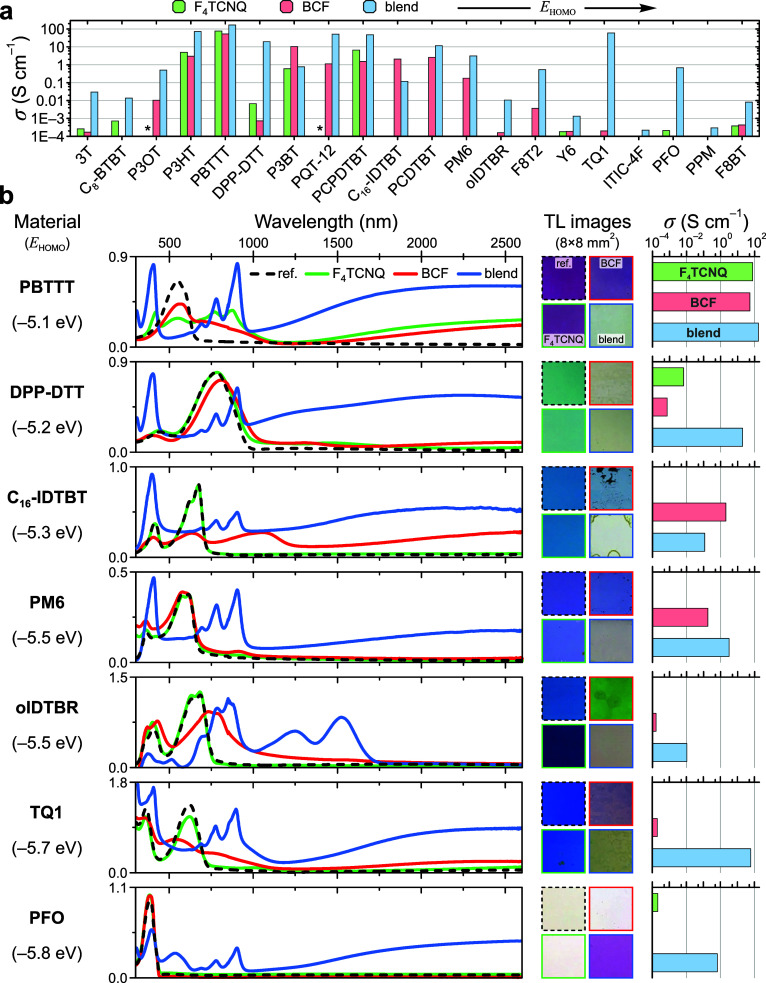
Generality of doping using Lewis-paired complexes. (**a**) Electrical conductivities σ obtained for the full range of studied OSCs arranged by increasing *E*_HOMO_ (left to right). (**b**) Summary of optical and electrical characteristics for a selection of benchmark OSCs, arranged by increasing *E*_HOMO_, showing UV-vis-NIR absorption spectra, transmitted-light (TL) images of ∼8 × 8 mm^2^ film regions and electrical conductivities. Data is shown for neat reference (“ref”) thin films, and the same films doped with BCF, F_4_TCNQ and 10:1 wt/wt BCF:F_4_TNCQ blend. The asterisk (*) indicates F_4_TCNQ-doped samples for which data is not available. In both panels, σ is reported for the as-doped, unoptimized samples.

With singular exceptions, doping with BCF:F_4_TCNQ yields markedly higher conductivities than those obtained using neat constituent dopants (e.g., PBTTT, DPP-DTT and PCPDTBT). Elsewhere, blend-doping is capable of inducing high conductivities (e.g., 100 and 1 S cm^–1^ for TQ1 and PFO respectively) in materials for which the individual dopants yield negligible values ≤10^–4^ S cm^–1^. In fact, blend-doping is also found to induce appreciable electrical conductivities in poly(phenylene methylene)^38,39^—a non-π-conjugated polymer with homoconjugation occurring along the backbone (Figure S18)—and nonfullerene acceptors (NFAs). Interestingly, the polaron signature in the absorption spectra of blend-doped NFAs (Figure 4b and Figure S19) is distinctly different to the observations for polymeric semiconductors and is likely to be related to restricted polaron delocalization for these small-molecular hosts.^32,40,41^

Further enhancement of electrical characteristics requires a better understanding of the doping process by Lewis-paired complexes and how it affects the microstructure of the host OSC. Figure 5a shows the IR spectra for a P3HT/BCF:F_4_TCNQ (quasi-)bilayer recorded in situ during progressive heating from 40 to 124 °C under N_2_. Thermal annealing leads to the appearance of a polaron background which extends across the entire spectral window and increases with temperature. Simultaneously, the peak at 2296 cm^–1^ ascribed to the B···N stretching mode, *υ*(B–N), increases in intensity and shifts toward lower frequencies depending on the degree of complexation and the ionization state (i.e., anion or dianion).^28,42^ By fitting these spectral features and plotting the polaron absorption intensity as a function of the *υ*(B–N) position (Figure 5b), we distinguish three doping regimes. (i) Below 80 °C, polaron formation and the associated electrical conductivity increase are predominantly due to the individual dopants, as inferred from the low *υ*(B–N) peak intensity and the characteristic F_4_TCNQ anion features visible in the IR (*υ*(C≡N) peak at 2187 cm^–1^) and UV-vis-NIR regions (Figure S20). (ii) Between 80 and 110 °C, the B···N peak shifts to lower frequencies, indicating BCF:F_4_TCNQ complexation which, assuming negligible dopant sublimation, is further corroborated by the bleaching of peaks at 2227 and 2187 cm^–1^ (neutral and anion forms of F_4_TCNQ, respectively). The absence of the 2187 cm^–1^ peak for the annealed samples furthermore suggests negligible formation of HF_4_TCNQ^–^ in a side-reaction,^13^ instead highlighting the preferential temperature-activated formation of the BCF:F_4_TCNQ complex. The electrical conductivity reaches a maximum within this region, with plausible variations due to the specific doping level and the degree of disorder introduced into a given OSC host. (iii) For temperatures >110 °C, polaron absorption intensity increases at a markedly different rate and its spectral center shifts toward higher frequencies, accompanied by a reduction of electrical conductivity. Notably, the B···N peak shifts completely to 2292 cm^–1^, indicating that the BCF:F_4_TCNQ complex exists predominantly in the “dianion” form, while the charges on the P3HT backbone are mostly bipolarons, as confirmed by electron paramagnetic resonance (EPR) spectroscopy (Figure S21).^28^ Hence, maximizing the electrical conductivity requires a precise control of the thermal process used to dope the material or, as advanced in this work, employing a subsequent solvent-based extraction step to partially and controllably dedope the material.

**Figure 5:**
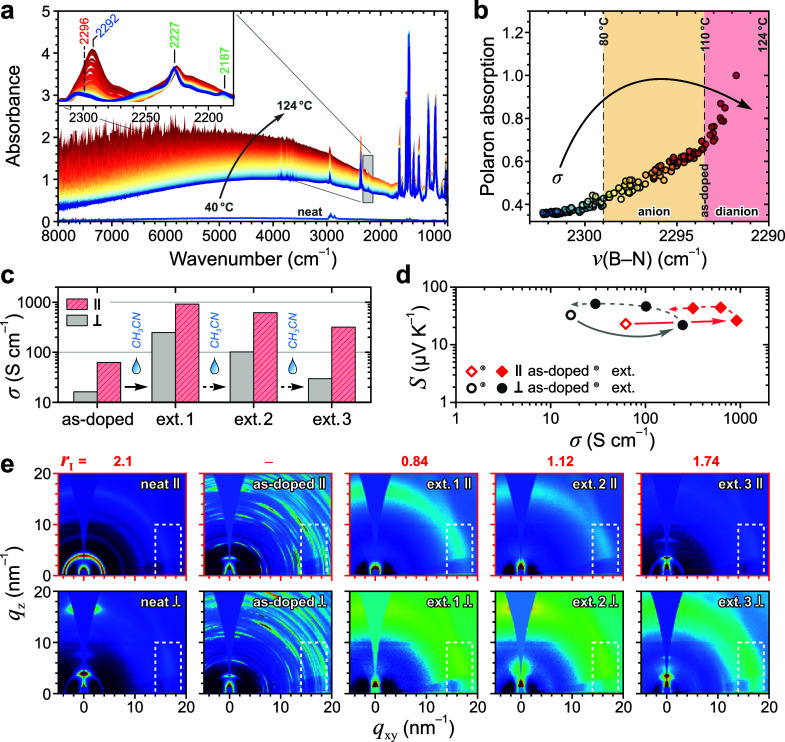
Maximising electrical conductivity via microstructure optimization. (**a**) IR spectra for P3HT/BCF:F_4_TCNQ bilayer film recorded in situ during heating from 40 to 124 °C at 2 °C min^–1^. Inset shows spectral features related to B···N and C≡N stretching modes after subtraction of the polaron background. (**b**) Normalized polaron absorption extracted from fitted spectra as a function of the B···N stretching mode position. The arrow provides a schematic depiction of the accompanying evolution of electrical conductivity. (**c**) Electrical conductivity for blend-doped oriented P3HT films measured parallel and perpendicular to the chain orientation axis, showing data for the as-doped sample and following sequential extraction steps with acetonitrile (‘ext.’). (**d**) Compiled Seebeck coefficient *S* and electrical conductivity values for the samples in (c). Solid arrows highlight the changes after the first extraction step; dotted arrows denote subsequent extraction steps. (**e**) 2D GIWAXS patterns for the identically fabricated oriented P3HT films measured with the beam aligned parallel (upper panel) and perpendicular (lower panel) to the film coating direction (i.e., the chain orientation axis). Data is shown for neat and blend-doped films: as-doped and following solvent extraction. The corresponding dichroic ratio (*r*_I_) values for the (0*k*0) reflections are indicated.

The above-mentioned solvent-based extraction method is illustrated for directionally oriented, anisotropic P3HT films fabricated by gas-assisted blade-coating of solutions comprising a crystallizable cosolvent.^5,43^ (*N.B*. Additional solvent-extraction data for in-plane isotropic blend-doped P3HT films reaching a conductivity of 110 S cm^–1^, shown in Figure S21, confirms the generality of this method.) Figures 5c–e summarize the analysis of interplay between microstructure and electrical characteristics for neat and blend-doped P3HT films, as well as the solvent-extracted (‘ext.’) blend-doped films obtained following successive spin-off treatments with acetonitrile to remove excess dopant.

GIWAXS patterns were recorded with the incident X-ray beam oriented parallel (ϕ_∥_) and perpendicular (ϕ_⊥_) to the film coating direction which, by virtue of the adopted fabrication, corresponds to the chain orientation axis (Figure 5e). The advantages of this approach are 2-fold: (i) it provides insights into the location and orientation of dopant molecules, and (ii) it allows to quantify the differences in structural disorder at the polymer backbone by comparing the dichroic ratio (*r*_I_) of the normalized (0*k*0) reflections at different doping levels.^44^ Here, neat P3HT film exhibits *r*_I_ = 2 and an isotropic distribution of grain orientations along ϕ_∥_ ascribed to the perturbation growth of lamella during the epitaxial crystallization process underpinning oriented film formation.^45^ Along ϕ_⊥_, the isotropic texture disappears and instead exhibits a bimodal distribution of grain orientations. Also noteworthy is that up to three orders can be observed for the (*h*00) peaks, indicating increased crystallinity in comparison to the conventional, in-plane isotropic blade-coated P3HT films.

Upon doping (“as-doped” samples), the (*h*00) scattering peaks shift toward lower scattering vectors for both ϕ_∥_ and ϕ_⊥_, indicating an expansion of the lamellar spacing and pointing to dopant allocating preferentially within the side-chains of the polymer network (Figure 5e and Figure S22). Polarized spectroscopic IR mapping supports this claim and furthermore suggests that the BCF:F_4_TCNQ dopant complex is oriented with its long axis perpendicular to the polymer backbone (Figures S23). The first solvent extraction step (‘ext. 1’) performed to remove the excess dopant reveals that dopant incorporation has introduced significant disorder along the π–π and lamellar directions. In particular, *r*_I_ decreases to less than half of the value for the neat material, while the (*h*00) and (0*k*0) scattering peaks are broadened and strongly attenuated. Subsequent repeated solvent extraction steps (‘ext. 2’ and ‘ext. 3’) yield partial recovery of the crystalline microstructure, additionally evidenced by the progressive increase of *r*_I_ (Figure S22). Further analysis is complicated by the additional scattering peaks ascribed to the presence of residual dopant on the film surface.

However, a delicate trade-off is found between microstructural order and doping level, as the corresponding electrical conductivity reaches a maximum after only the first extraction step, decreasing with each subsequent dedoping treatment (Figure 5c). The primary role of microstructural optimization underpinning the increase of conductivity following solvent-based extraction can be additionally inferred from its correlation with the respective Seebeck coefficient (*S*) values. As shown in Figure 5d, while the first solvent extraction step dramatically increases σ values by over an order of magnitude—up to 915 and 247 S cm^–1^ parallel and perpendicular to the P3HT chain orientation axis —the corresponding *S* values remain essentially constant at 26 ± 4 μV K^–1^. Seebeck coefficient is known to exhibit a strong inverse relation with electrical conductivity (*S* ∝ σ^–1/4^),^46,47^ depending primarily on charge-carrier concentration and being essentially invariant with carrier mobility. Given the above, as well as previous reports on oriented and doped P3HT films,^48−50^ the data suggest that judicious solvent-based dedoping primarily enhances microstructure-dependent charge-carrier mobility without significantly affecting carrier concentration. Overall, the results in Figure 5 highlight that the key to maximizing electrical conductivity of blend-doped films is maintaining a high doping level while reducing the microstructural disorder arising due to the incorporation of large dopant counterions. In summary, the thermoelectric power factor for P3HT doped with the blend is ca. 10 times higher than any of the neat dopants for isotropic films, and up to 50 times larger for oriented films.

Finally, the studied Lewis-paired dopant complexes are reviewed to outline their application potential and benchmark their performance in terms of the resulting optical and electronic properties, processability and generality. BCF:F_4_TCNQ dopants yield state-of-the-art electrical conductivities, leading to near-complete bleaching of neutral OSC absorption in the visible spectral region (Figure 4b) while retaining negligible transmission haze.^51^ Hence, Lewis-paired dopants present an intriguing alternative for the fabrication of transparent conductive electrodes (TCEs). Estimates of Haacke’s figure-of-merit^52^ based on film transmittance at 550 nm and sheet resistance (FOM = *T*^10^/*R*_s_) show that blend-doped P3HT (Figure 1c) and PBTTT (Figure 4b) feature FOM values that are only a factor of 8–23 lower than classical solution-processed materials such as PEDOT:PSS (Table S1, Supporting Information). In terms of processability, BCF:F_4_TCNQ solutions are found to feature exceptional long-term stability in comparison to F_4_TCNQ solutions that are prone to rapid oxidation. Therefore, the demonstrated solution-sequential blend-doping process naturally lends itself to large-scale fabrication of high-resolution doping patterns for OFETs by inkjet printing^53^ or “molecular-gate”-based photothermal methods.^54^ Elsewhere, besides the use of BCF:F_4_TCNQ to dope a wide range of OSCs, the generality of Lewis-paired dopants additionally implies the use of other complex-forming small molecules, which we demonstrated by, e.g., substituting BCF with FeCl_3_ and F_4_TCNQ with its nonfluorinated analogue TCNQ (Figure S24). In addition to the reduction of material costs, this approach may be used to circumvent the disruption of microstructural order and reduced conductivity within doped OSCs by employing comparatively smaller molecules such as −CN-bearing TCNQ and malononitrile or Lewis acids such as BF_3_.

An additional comment is necessary on the differences between this work and the recent reports by Suh et al.^28^ and Mansour et al.^34^ These presented an in-depth examination of Lewis-paired dopant complexes and reached similar conclusions regarding the suitability of BCF:F_4_TCNQ for doping a range of OSC, as well as the improved thermal- and bias-stress stability. However, the maximal reported conductivities of 10–20 S cm^–1^ for solution-sequential doping,^28^ or even lower in the case of doping by codeposition,^34^ did not reflect the full potential of this novel dopant system. The more than 10-fold enhanced conductivities reported in this work (Figure 4a) are likely to have been enabled, in part, by developing a deeper understanding of the thermal process underpinning both the complex formation and doping (Figure 5a,b). While in the work of Suh et al. the as-doped materials were understood to exhibit the maximal doping level, we show instead that judicious control of thermal annealing—and solvent-based postprocessing—are required to optimize the electrical conductivities. In addition, while Suh et al. provisionally ascribed the limited conductivity values to microstructural disruption arising due to the use of a moderate solvent (dichloromethane) for solution-sequential doping, we show that this effect persists even for a fully orthogonal solvent (95:5 vol/vol acetonitrile:ethyl acetate). Hence, doping-induced disorder is proposed to be solvent-independent, thereby requiring careful material- and process selection to achieve maximal electronic performance.

In summary, Lewis-paired dopant complexes such as BCF:F_4_TCNQ are advanced as a uniquely promising and versatile p-dopant system for an exceptionally wide range of OSCs. State-of-the-art electrical conductivities are obtained even for classical materials such as P3HT, exceeding 300 and 900 S cm^–1^ for in-plane isotropic and chain-oriented films respectively. Appreciable conductivities are also obtained for NFAs and materials with deep-lying HOMO levels such as π- and homoconjugated polyfluorene (co)polymers and poly(phenylene methylene). The large size and high binding energy of BCF:F_4_TCNQ complexes furthermore ensure improved thermal stability of doped films, featuring, in the case of P3HT, 3-fold higher dedoping activation energies than those of the constituent dopants, as well as exceptionally high operational lifetime at 100 °C in air. While the incorporation of large Lewis-paired dopants has a tendency to disrupt the neat OSC microstructure, its detrimental effects can be effectively ameliorated by thermal or solvent-based fabrication adjustments. Finally, the R2R-compatible solution-based processing employed in this work, as well as cost-reduction strategies via dopant selection from a wide library of Lewis-pairing molecules, present numerous avenues for the industrial fabrication of organic electronic devices featuring a step-change improvement in both stability and performance.

## Supporting Information Available

Experimental and materials details; optical and IR/Raman spectroscopy analysis of doped OSC films and reference dopant-only films; thermal analysis of neat and blended dopants by DSC; DFT calculations; additional electrical conductivity and Seebeck coefficient data for doped P3HT films; optical spectroscopy and electrical conductivity for additional blend-doped OSCs; GIWAXS data; Haacke FOM calculation (PDF)
