# Supplementary material for: A Universal, Highly Stable Dopant System for Organic Semiconductors Based on Lewis-Paired Dopant Complexes
Source: ACS Energy Lett. 2024 Jul 1;9(7):3567–77. doi: 10.1021/acsenergylett.4c01278 (PMC11249766; doi:10.1021/acsenergylett.4c01278)
Supplement: Supplementary file 1 — nz4c01278_si_001.pdf [file nz4c01278_si_001.pdf]

Supporting Information

A Universal, Highly Stable Dopant System for Organic Semiconductors Based on Lewis-Paired Dopant Complexes

Osnat Zapata-Arteaga, Aleksandr Perevedentsev, Michela Prete, Stephan Busato, Paolo Sebastiano Floris, Jesika Asatryan, Riccardo Rurali, Jaime Martín, Mariano Campoy-Quiles**

* aperevedentsev@icmab.es
* mcampoy@icmab.es

1. Materials and methods

Materials.

Poly(3-hexylthiophene) (P3HT) was purchased from Merck (product no. 698997: M_{n} = 54–75 kDa, RR > 98% and product no. 445703: M_{w} = 68 kDa, RR > 98.5%) and Rieke Metals (M_{w} = 68 kDa, $M_{\mathrm{w}}/M_{\mathrm{n}}$ = 2.4, RR = 95%; used only for thermal stability analysis in Figure 3). Tris(pentafluorophenyl)borane (BCF) and 2,3,5,6-tetrafluoro-7,7,8,8-tetracyanoquinodimethane (F_4TCNQ) were purchased from TCI Chemicals and 1-Material respectively. Bis(trifluoromethane)sulfonimide lithium salt (LiTFSI) was purchased from Merck. Standard laboratory-grade solvents were used throughout. Additional material details are given in Section 2 below.

Fabrication.

All thin-film fabrication and doping was performed under ambient atmosphere, except pre-crystallisation of OSC films performed in N_2 glovebox. P3HT was blade-coated from solutions in chlorobenzene using gas-assisted drying[1] to obtain homogeneous large-area films, with similar processes adopted for all other OSC films. Dopants were dissolved in a 95:5 vol/vol acetonitrile : ethyl acetate mixture at 5 wt% (F_4TCNQ) and 10 wt% (BCF and BCF:F_4TCNQ). In all cases, sequential doping was performed by spin- (3000 rpm) or blade-coating (40 °C, 10 mm s^{-1} coating speed) dopant solutions onto pre-crystallised OSC films followed by 10 s thermal annealing in air at 100 °C (F_4TCNQ) and 120 °C (BCF and BCF:F_4TCNQ). Unless specified otherwise, 10:1 wt/wt (≈5:1 mol/mol) BCF:F_4TCNQ dopant blends were employed. Typical 'solvent extraction' process to remove excess dopant from the doped films involved spin- (100 µL, 3000 rpm) or blade-coating (40 °C, 100 µL, 5 mm s^{-1} coating speed) pure acetonitrile as the best solvent for all studied dopants. Directionally oriented P3HT films were fabricated by gas-assisted blade-coating of P3HT solutions in chlorobenzene:1,3,5-trichlorobenzene (TCB) as described in the literature.[1,2]

Characterisation.

All analyses were performed under ambient atmosphere unless noted otherwise.

Optical spectroscopy: UV-Vis-IR absorption spectra were measured using a Jasco V780 spectrophotometer without reflection correction. Polarised Vis-NIR absorption spectra were recorded with a Bruker Vertex 70 FTIR spectrophotometer coupled to a Bruker Hyperion optical microscope.

GIWAXS analysis: Measurements were performed at the NCD-SWEET beamline at the ALBA synchrotron (Spain). The scattering experiments were performed in grazing-incidence geometry, using a 50-µm-diameter beam and energy of 12.4 keV, with the Pilatus detector at a distance of 21 cm. The incidence angle of the X-ray radiation was fixed at 0.12° with respect to the sample plane for all the experiments, and an acquisition time of 1 s was used.

IR spectroscopy: In-situ IR spectroscopy of thermally annealed samples and spectroscopic mapping were performed under N_2 blanket at the BL01-MIRAS beamline of ALBA synchrotron. Additional IR spectroscopy was performed using a Bruker Vertex 70 FTIR spectrophotometer coupled to a Bruker Hyperion optical microscope.

Raman spectroscopy: Raman spectra were recorded using a WITec Alpha 300RA instrument. Raman spectroscopy was performed using a WITec Alpha300RA instrument, with the typical spectra recorded

over ~3×3 mm^2 sample areas in the scanning mode, followed by the averaging of data from ~900 points and subtraction of a polynomial background. No sample dedoping or degradation was observed under the adopted conditions. Polarized Raman spectra were recorded with both excitation and detection polarized collinearly, and using constant excitation, detection, and sensitivity settings throughout.

Thermal analysis: BCF:F_4TCNQ powder mixtures were prepared and stored under N_2 atmosphere immediately prior to measurements. DSC was performed on 6–16 mg of blended materials hermetically sealed inside standard aluminium pans to avoid material sublimation altering the blend composition during thermal cycling. DSC pans were weighed before and after measurement to confirm negligible weight loss. 5 °C min^{-1} heating/cooling rates were used throughout. TGA was performed on material in open pans under N_2 flow using 5 °C min^{-1} heating rates. The higher heating rate in comparison to the more conventional 2 °C min^{-1} was selected to minimize excessive sublimation of BCF during heating while allowing for any complex formation.

Electronic structure calculations: We performed density-functional theory (DFT) calculations with the VASP code[3] and projector augmented waves,[4,5] using an energy cutoff of 400 eV and the generalised-gradient approximation[6] for the exchange-correlation energy. In each self-consistency cycle the charge density is considered to be converged when the total energy change and the band-structure-energy change between two steps are both smaller than 10^{-7} eV. The atomic positions were optimised until all the atomic forces were smaller than 0.02 eV $Å^{-1}$ using a quasi-Newton algorithm. Energy levels are referred to the vacuum energy, obtained by computing the total local potential and tracking its evolution along the three coordinate directions (and taking the average value). Additional DFT calculations of IR spectra in the gas state were done with the ORCA package, with both the optimisation and frequency calculations performed using a hybrid functional BP86 and basis set DEF2-SVP.

Others: Electrical conductivity was measured in the Van der Pauw configuration using a custom-built setup described elsewhere.[7] EPR measurements were performed on a Bruker ELEXYS E500 X band EPR spectrometer. Optical microscopy was performed using an Olympus BX51 instrument.

2. Additional materials

- C8-BTBT (CAS No. 583050-70-8): obtained from Merck.
- PBTTT (‘C14-PBTTT’; CAS No. 888491-19-8): obtained from 1-Material.
- P3OT (regioregular; CAS No. 104934-51-2): obtained from Rieke Metals.
- P3BT (regioregular; CAS No. 98837-51-5): obtained from Merck.
- DPP-DTT (CAS No. 1260685-66-2): obtained from 1-Material.
- PM6 (‘PBDB-T-2F’; CAS No. 1802013-83-7): obtained from Solarmer.
- o-IDTBR (CAS No. 2077945-91-4): obtained from 1-Material.
- Y6 (‘BTP-4F’; CAS No. 2304444-49-1): obtained from 1-Material
- TQ1 (CAS No. 565228-37-7): obtained from Ossila.
- PFO (M_W = 77 kDa, PDI = 2.65; CAS No. 19456-48-5): obtained from Ossila.
- Terthiophene (‘3T’; CAS No. 1081-34-1): obtained from TCI Chemicals.
- PQT-12 (CAS No. 827343-06-6): obtained from Merck.
- PCPDTBT (M_W = 67 kDa, PDI = 2.09; CAS No. 920515-34-0): obtained from Ossila.
- MDMO-PPV (CAS No. 177716-59): obtained from Sigma-Aldrich.
- PCDTBT (M_W = 43 kDa, PDI = 2.27; CAS No. 958261-50-2): obtained from Ossila.
- F8T2 (M_W = 116 kDa, PDI = 2.6; CAS No. 210347-56-1): obtained from Ossila.
- ITIC-4F (CAS No. 2097998-59-7): obtained from 1-Material.
- Poly(phenylene methylene) (‘PPM’, M_W = 36 kDa, PDI = 3.3): synthesised by the group of Prof. Walter R. Caseri, Department of Materials, ETH Zürich, Switzerland.
- F8BT (M_W < 25 kDa; CAS No. 210347-52-7): obtained from Merck.

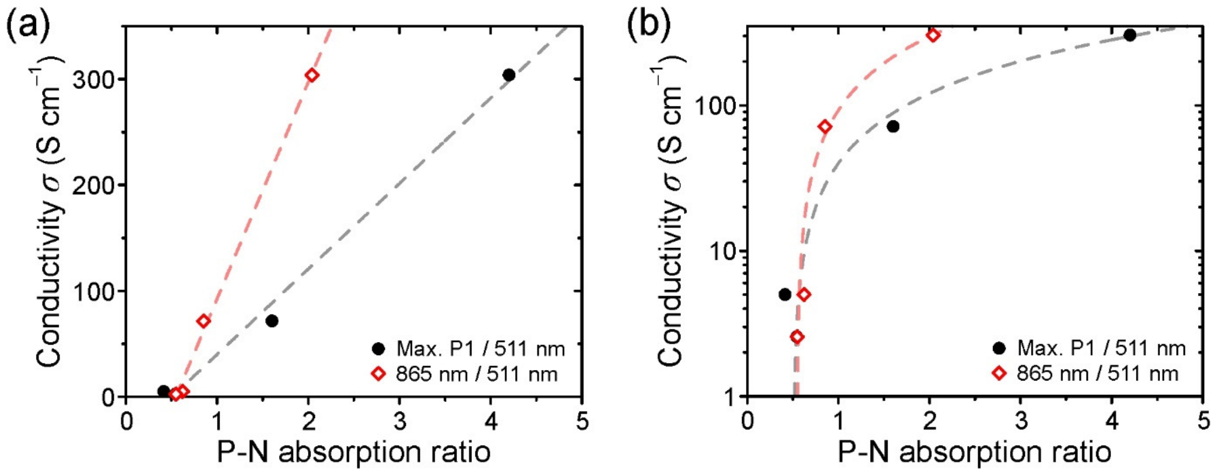

Figure S1. Electrical conductivity σ plotted as a function of polaron (P) to neutral polymer (N) absorption ratio on (**a**) linear and (**b**) semi-logarithmic scale. Data is extracted from absorption spectra of P3HT doped with BCF, F4TCNQ and BCF:F4TCNQ (Fig. 1c in the main text). In each case, absorption ratios correspond to the maximum absorbance of the P1 band (1100–2650 nm) (●) or absorbance at 865 nm (approximately corresponds to the P2 band) (◇) relative to absorbance at 511 nm (neutral polymer). Dashed lines represent linear fits and guides to the eye in (a) and (b) respectively.

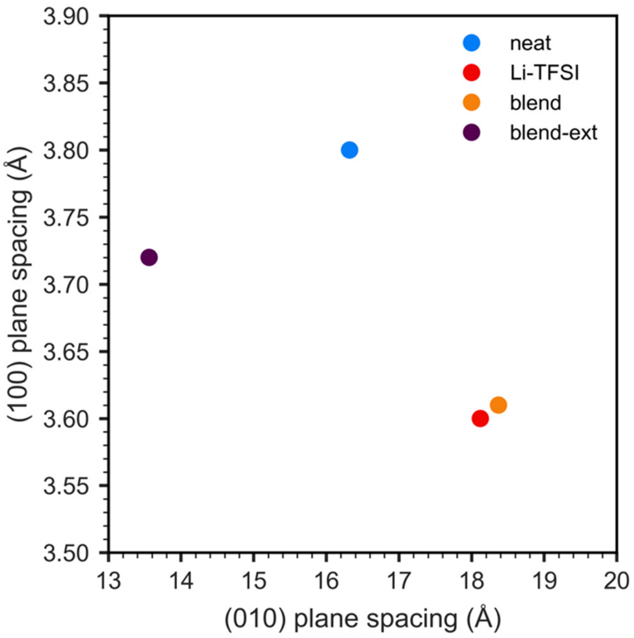

Figure S2. Plane spacings calculated for the (100) and (010) reflection peaks for neat and doped P3HT films from GIWAXS analysis. The presented data is an average from several 2D-GIWAXS patterns recorded at different incident angles, and their corresponding linecuts.

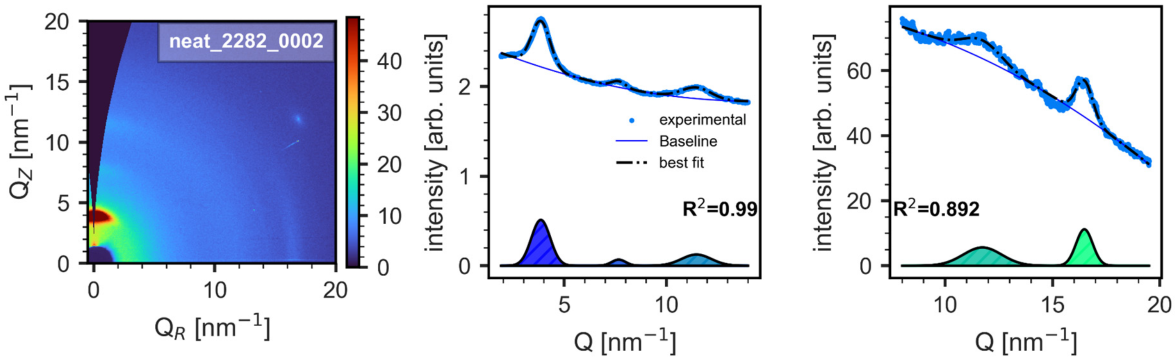

	center	height	sigma	amplitude	fwhm	d-space
p(100)	3.85	800.0	0.462	0.592	1.088	16.32
p(200)	7.664	10.0	0.344	0.059	0.81	8.198
p(300)	11.474	4.0	0.728	0.228	1.714	5.476
p(003)	11.739	10.0	0.897	12.612	2.112	5.353
p(010)	16.486	20.0	0.387	10.861	0.912	3.811

Figure S3. Summary of GIWAXS analysis for a neat, undoped P3HT film. 2% of the background was subtracted for clarity.

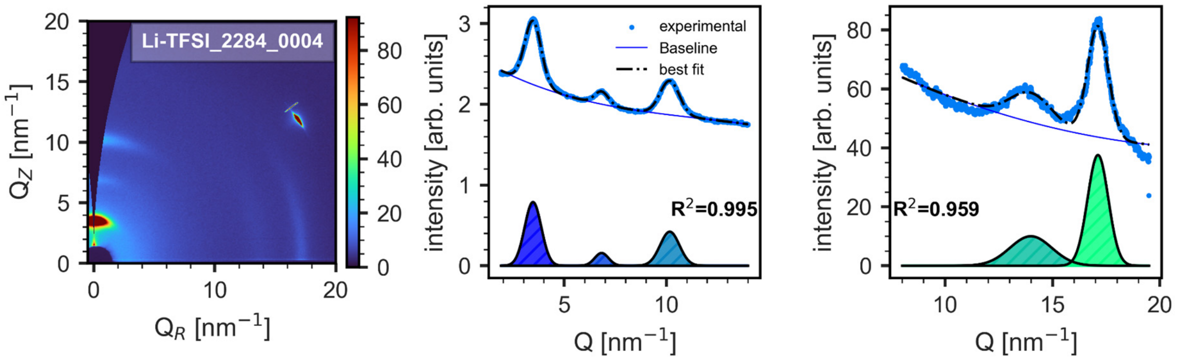

	center	height	sigma	amplitude	fwhm	d-space
p(100)	3.466	800.0	0.408	0.806	0.96	18.126
p(200)	6.827	10.0	0.337	0.133	0.794	9.203
p(300)	10.168	4.0	0.473	0.499	1.114	6.18
p(003)	13.994	10.0	0.976	24.447	2.297	4.49
p(010)	17.118	20.0	0.484	45.53	1.139	3.671

Figure S4. Summary of GIWAXS analysis for a reference LiTFSI-doped P3HT film. 2% of the background was subtracted for clarity.

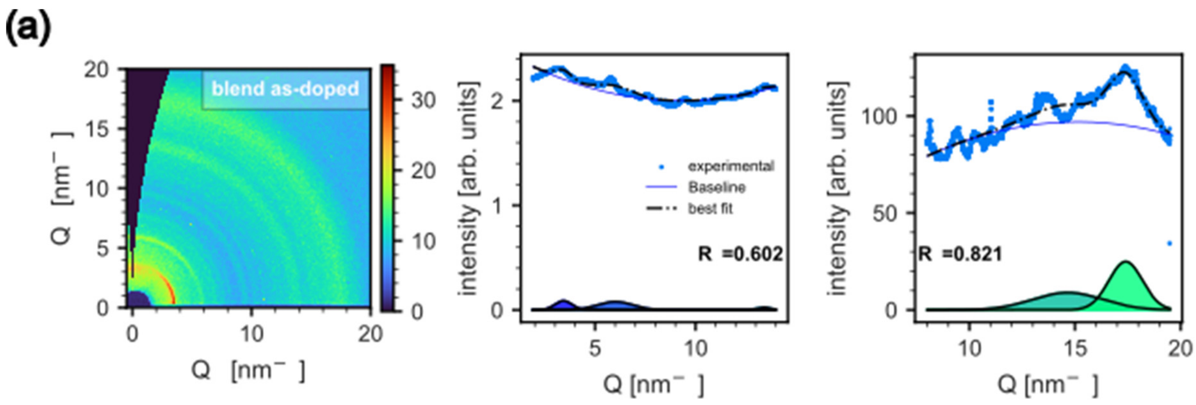

	center	height	sigma	amplitude	fwhm	d-space
p(100)	3.42	800.0	0.425	0.093	1.002	18.371
p(200)	6.0	10.0	0.888	0.177	2.09	10.472
p(300)	13.402	4.0	0.35	0.023	0.824	4.688
p(003)	14.654	10.0	1.663	37.329	3.917	4.288
p(010)	17.403	20.0	0.8	50.0	1.883	3.61

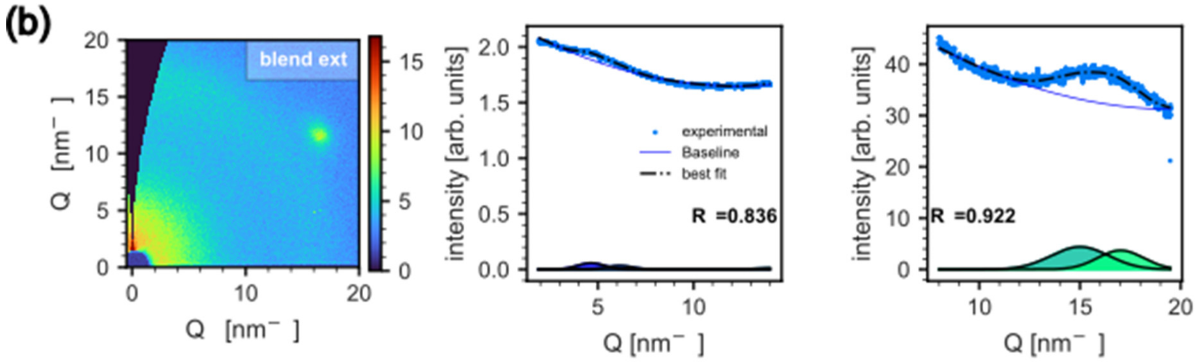

	center	height	sigma	amplitude	fwhm	d-space
p(100)	4.644	800.0	0.72	0.105	1.696	13.529
p(200)	6.134	10.0	0.701	0.061	1.652	10.242
p(300)	13.955	4.0	0.693	0.025	1.632	4.503
p(003)	15.0	10.0	1.435	15.863	3.379	4.189
p(010)	17.017	20.0	1.138	10.597	2.68	3.692

Figure S5. Summary of GIWAXS analysis for BCF:F_4TCNQ-doped P3HT films: (**a**) as-doped and (**b**) following solvent-based extraction. 2% of the background was subtracted for clarity.

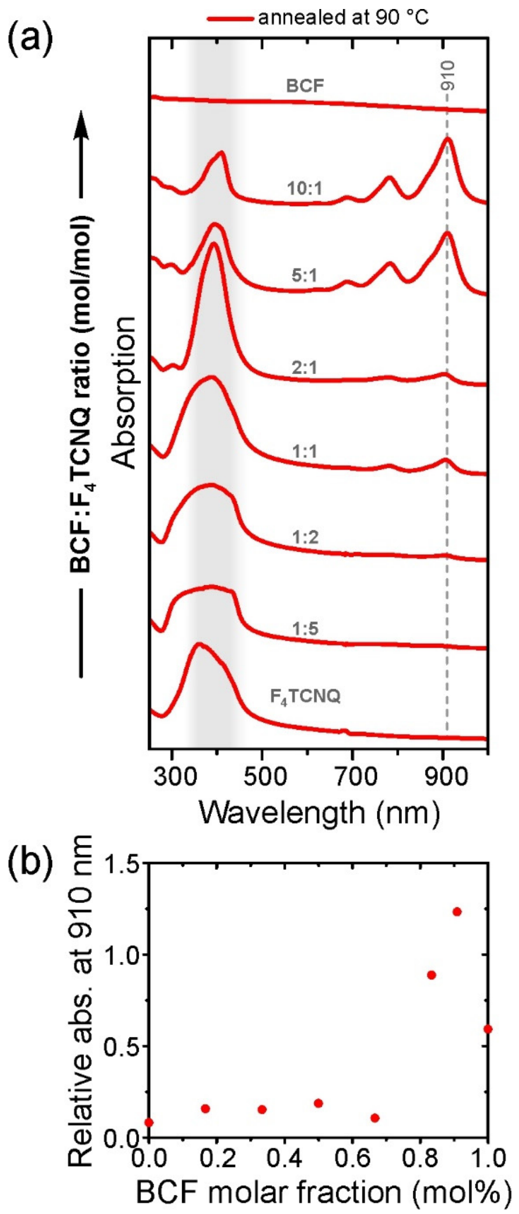

Figure S6. (**a**) UV-Vis-NIR (not normalised) spectra of BCF:F_4TCNQ blend films at varying molar compositions, recorded following thermal annealing at 90 °C. (**b**) Absorption at 910 nm relative to maximum absorption in the 350–420 nm spectral region (shaded area in (a)) as a function of BCF molar fraction.

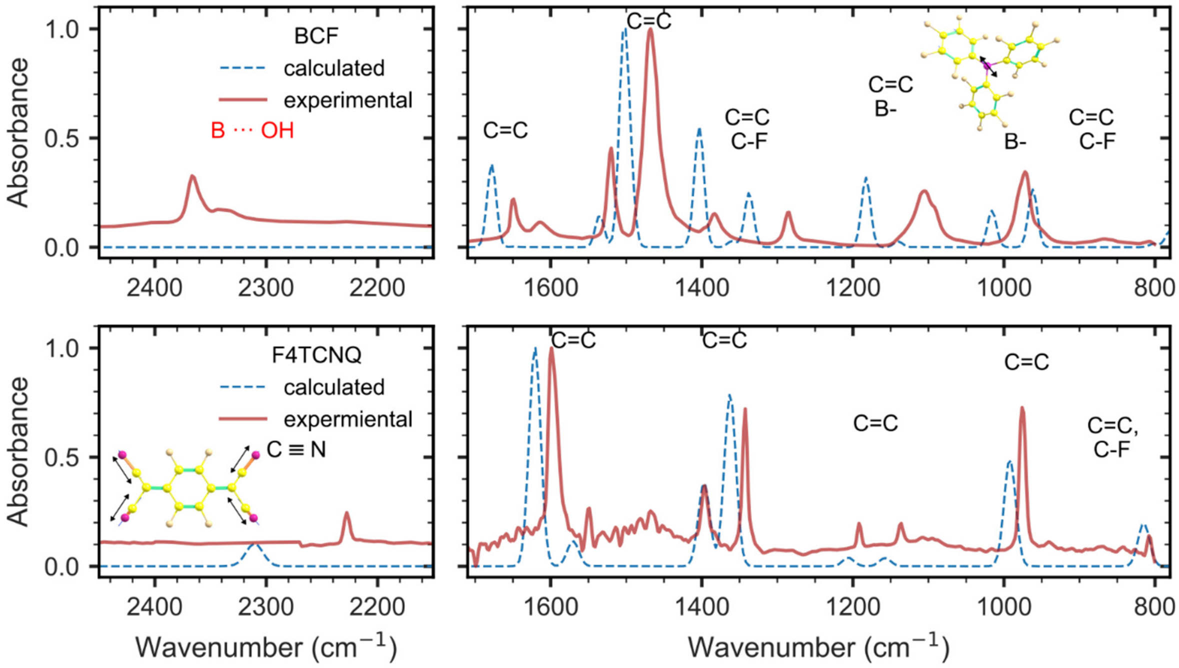

Figure S7. IR spectra of BCF (*upper panel*) and F_4TCNQ (*lower panel*), showing both DFT-calculated and experimentally measured spectra.

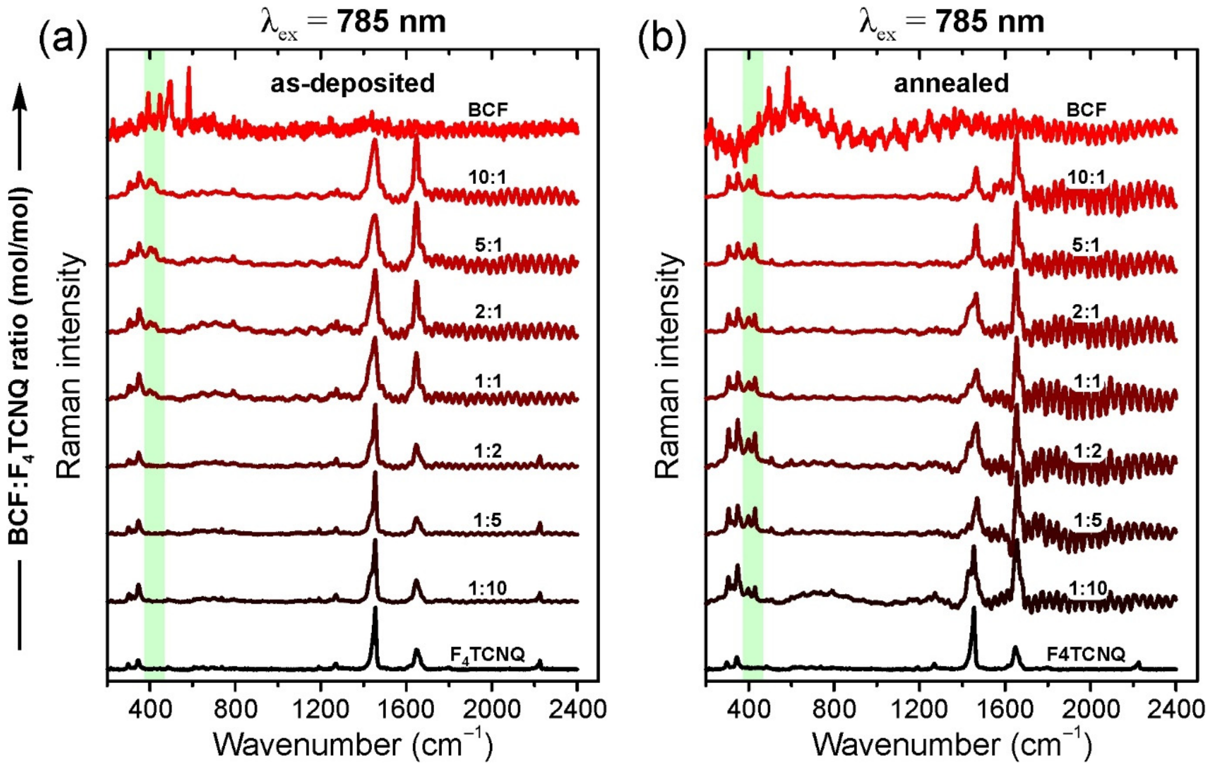

Figure S8. Comparison of Raman spectra recorded using excitation at 785 nm for (**a**) as-deposited and (**b**) annealed BCF:F_4TCNQ blend films. Respective molar ratios are indicated; all spectra are peak-normalised for clarity. Shaded areas highlight spectral regions where new peaks appear following thermal annealing.

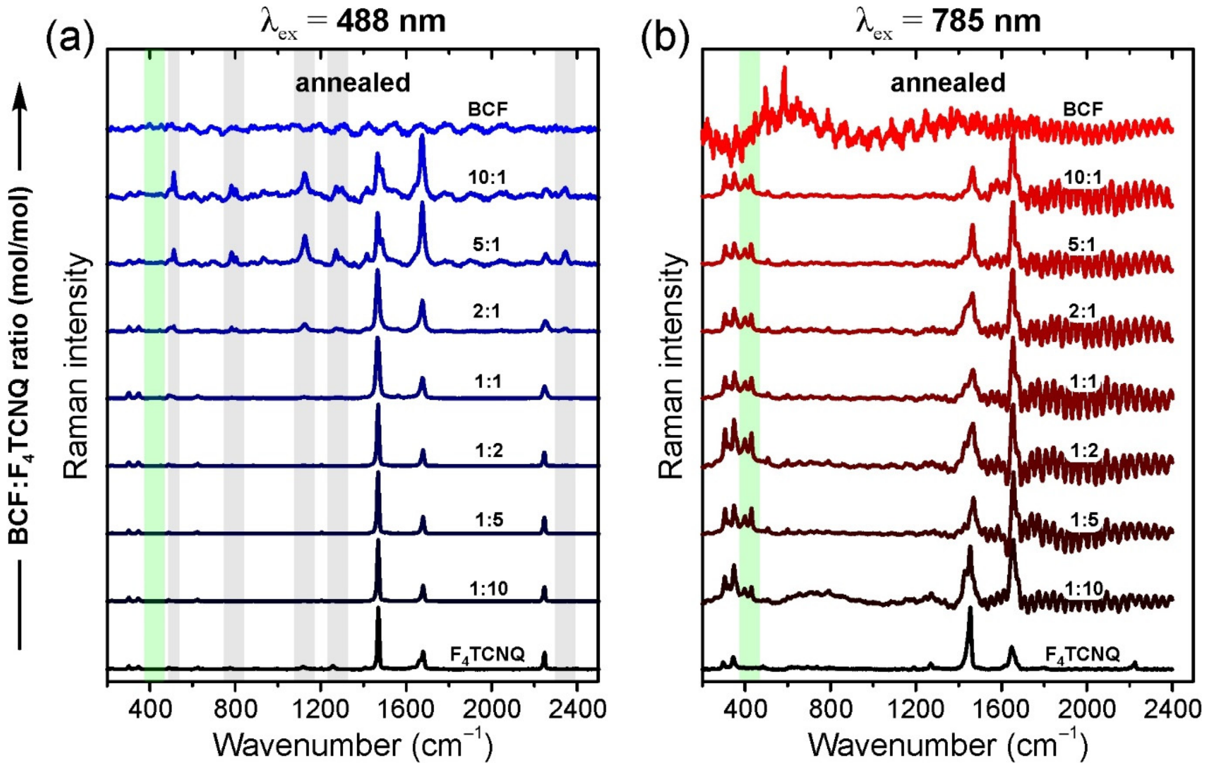

Figure S9. Comparison of Raman spectra recorded for annealed BCF:F_4TCNQ blend films using excitation at (**a**) 488 and (**b**) 785 nm. Respective molar ratios are indicated; all spectra are peak-normalised for clarity. Shaded areas highlight spectral regions where new peaks appear upon increasing the BCF molar fraction in the blends.

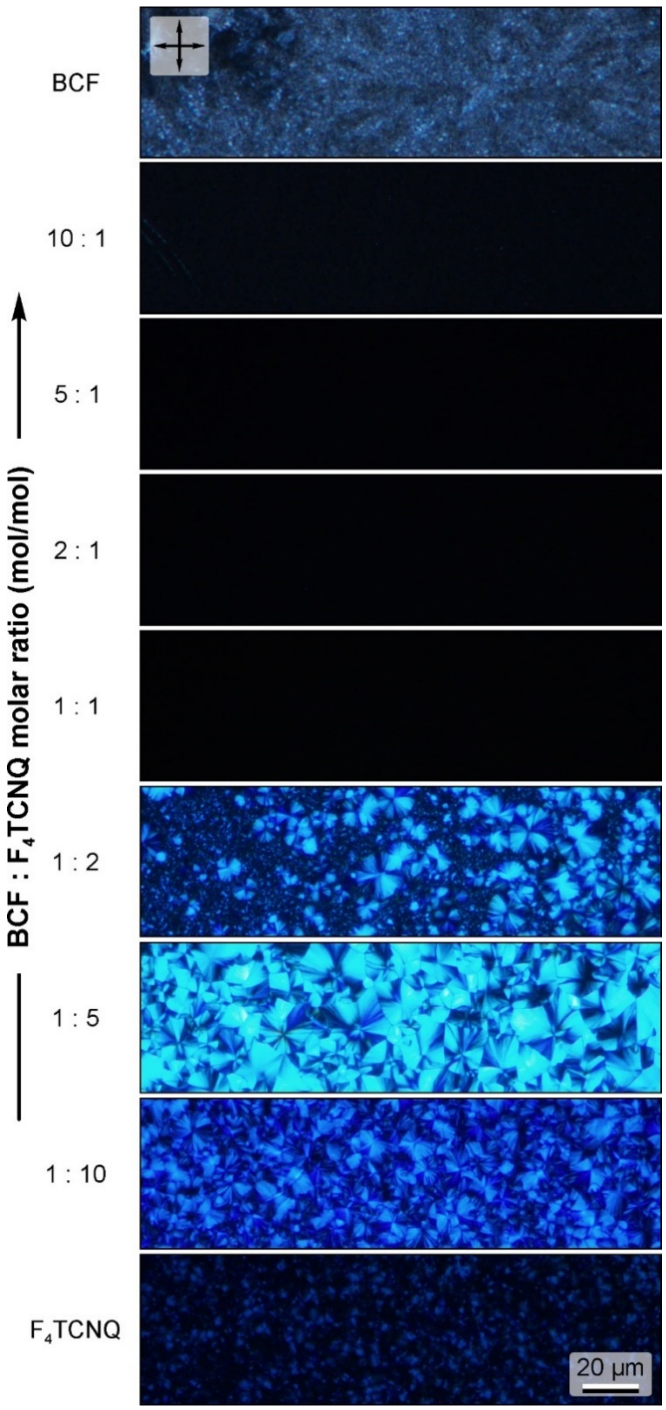

Figure S10. Cross-polarised micrographs of BCF:F_4TCNQ blend films as a function of increasing BCF molar fraction recorded following annealing at 90 °C. Identical image acquisition settings were used. The increased crystallite size at low BCF:F_4TCNQ molar ratios (1:10 – 1:2 mol/mol) in comparison to neat F_4TCNQ is indicative of BCF acting as a diluent for F_4TCNQ. Vitrification of the blends at moderate-to-high BCF:F_4TCNQ molar ratios (1:1 – 10:1 mol/mol) suggests that, in contrast to the neat dopants, the BCF:F_4TCNQ complex is largely amorphous.

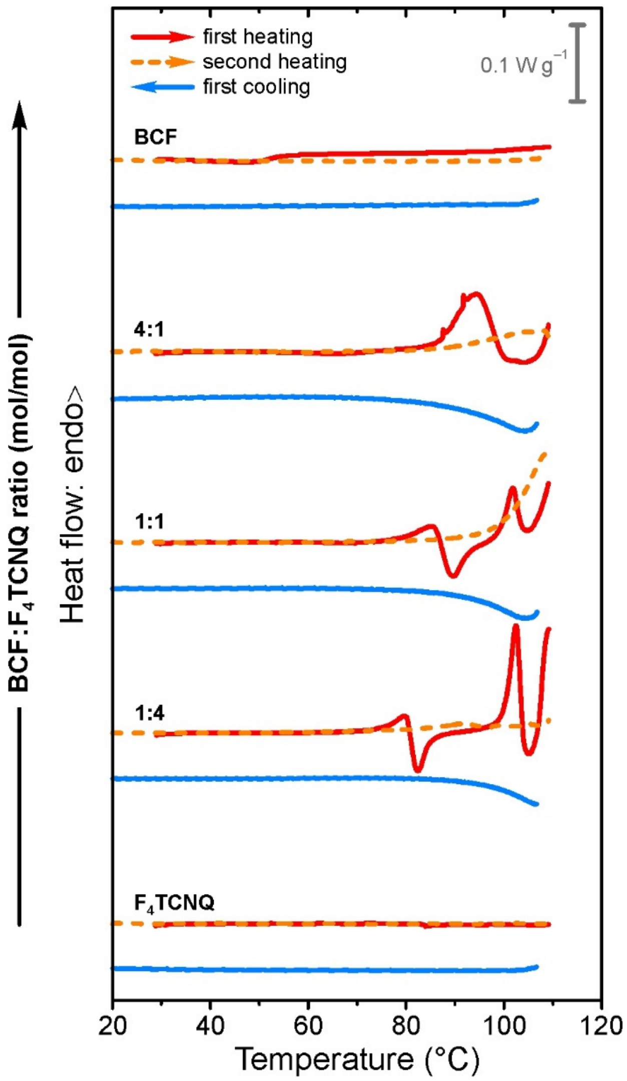

Figure S11. DSC thermograms for BCF, F_4TCNQ and blends thereof, showing first- and second-heating and first-cooling traces. Thermograms are presented for selected BCF:F_4TCNQ molar compositions (mol/mol ratios are indicated) and are offset for clarity. Heat flow is normalised by the total mass of the materials (cf. W g^{-1} scale bar).

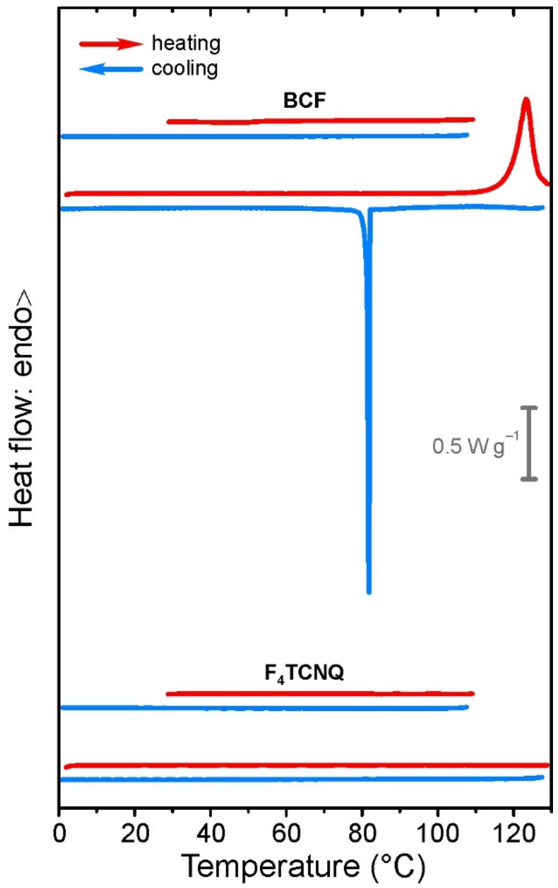

Figure S12. Thermal analysis for neat BCF and F_4TCNQ. Heating and cooling DSC thermograms are shown for a standard (*upper datasets*) and extended (*lower datasets*) temperature range. Thermograms are offset for clarity; heat flow is normalised by the total mass of the materials (cf. W g^{-1} scale bar). Extended-range thermal cycling displays the melting endotherm of BCF during heating and a sharp crystallisation exotherm upon subsequent cooling. F_4TCNQ thermograms are featureless in all cases.

Differential scanning calorimetry (DSC) was used to identify signatures of BCF:F_4TCNQ complex formation. Dry-mixed powder blends were used to avoid any complex formation simultaneous with exposure to solvents. The first-heating thermograms for the blends (**Figure S11**) display a series of overlapping endo- and exothermal transitions in the 75–110 °C range, consistent with the thermal post-processing used for doping. The transitions are *irreversible*, with the subsequent first-cooling and second-heating thermograms being essentially featureless. In contrast, all thermograms for the neat dopants (**Figure S12**) are featureless across the entire 20–110 °C range. (*N.B.* Extending the range up to 130° C for BCF reveals thermal transitions with a substantially different signature, namely *reversible* melting and crystallisation transitions at 123 and 82 °C respectively; see **Figure S12**.) Hence, the transitions observed in the first-heating thermograms of dopant blends are ascribed to the BCF:F_4TCNQ complex formation via a *monotropic solid–solid transition*, i.e. without entering a melt/liquid phase. This assignment is corroborated by the irreversibility of the transitions and their comparatively low enthalpy, whereby peak heat-flows observed in first-heating thermograms of dopant blends are at least 5-fold lower than for melting/crystallisation transitions of neat BCF (cf. heat-flow scale bars in **Figure S11** and **Figure S12**). Furthermore, it was confirmed that the samples remained in powder form following DSC thermal cycling.

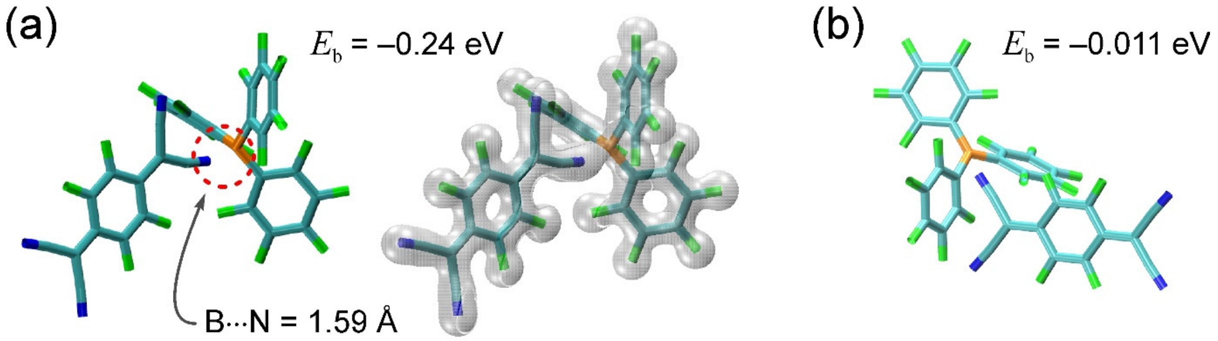

Figure S13. Binding geometries of the BCF:F_4TCNQ complex, showing (**a**) the stick representation and charge density distribution for the most stable complex geometry (the distance between B and N atoms is highlighted) and (**b**) a less stable complex geometry. The corresponding binding energies, E_b, are indicated.

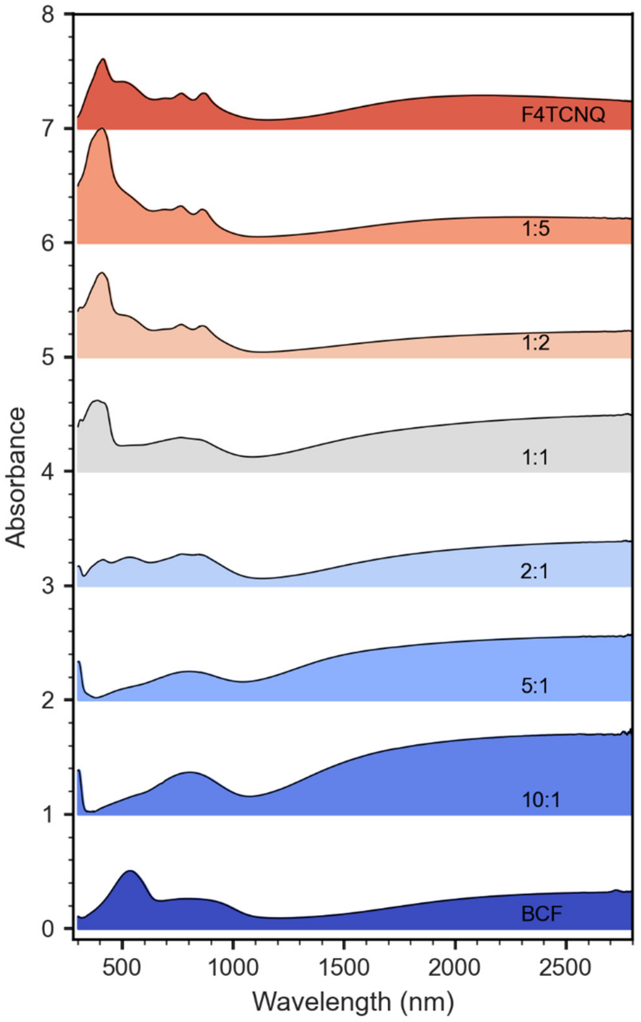

Figure S14. UV-vis-NIR spectra of P3HT films doped with BCF:F_4TCNQ blended at varying molar ratios (BCF:F_4TCNQ mol/mol values indicated). The spectra are offset for clarity.

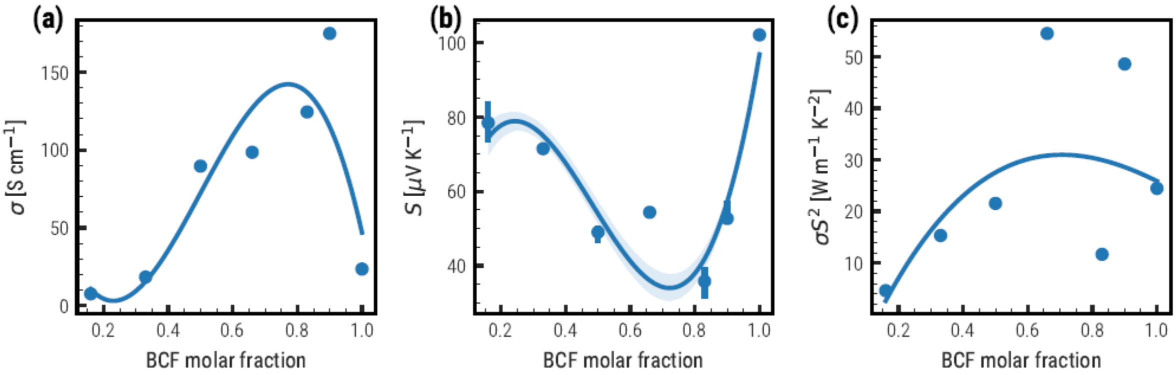

Figure S15. Electrical and thermoelectric characteristics of P3HT films doped with BCF:F_4TCNQ blended at varying molar ratios: (**a**) electrical conductivity, (**b**) Seebeck coefficient and (**c**) power factor. Error bars are obtained from four measurements performed on the same sample.

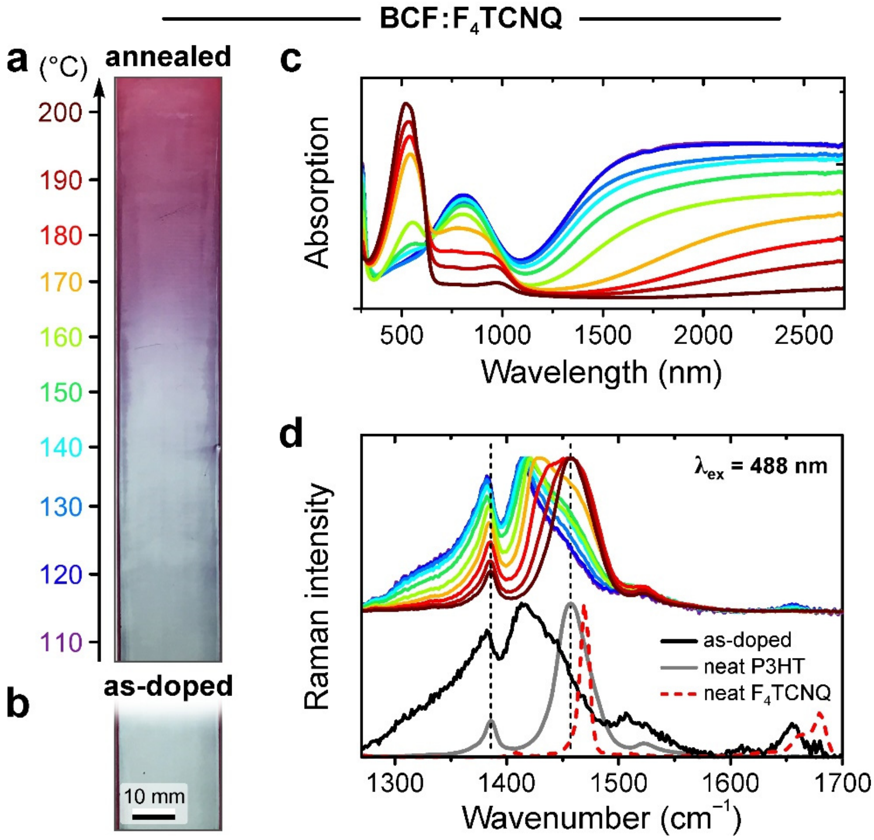

Figure S16. Thermal dedoping of P3HT films doped 10:1 mol/mol BCF:F_4TCNQ blend. (**a**) Transmitted-light images of doped P3HT films following thermal annealing along a temperature gradient spanning 110–200 °C across the substrate for 15 min in air and (**b**) section of the same film prior to annealing. (**c**) Absorption spectra for the doped film shown in (a) as a function of annealing temperature. (**d**) Raman spectra (λ_{ex} = 488 nm) for the BCF:F_4TCNQ-doped film as a function of annealing temperature (*top panel*) and reference spectra for the as-doped film, as well as neat P3HT and F_4TCNQ.

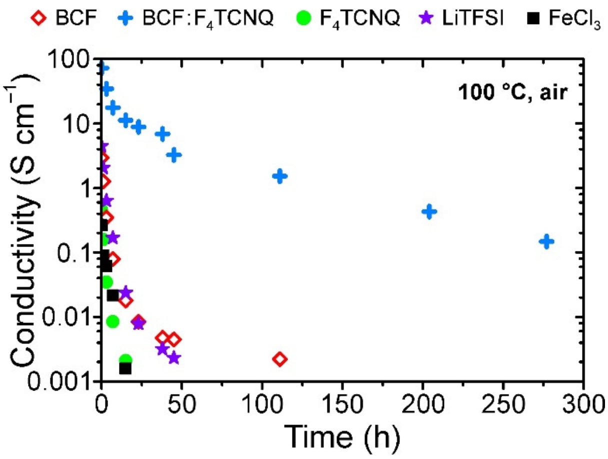

Figure S17. 'Operational' stability of doped P3HT films, showing electrical conductivity as a function of prolonged thermal annealing at 100 °C in air.

P3HT films (film thickness = 190 nm) were pre-crystallised at 150 °C and doped as follows:

- **BCF:** 10 wt% solution in methanol was blade-coated (40 °C; 50 mm s^{-1}) onto P3HT films and annealed at 120 C for 10 s, repeating this process one more time to ensure uniform doping of the relatively thick P3HT films. Excess dopant was removed by spin-off with 50 µL of acetonitrile at 3000 rpm.
- **BCF:F_4TCNQ:** 15 wt% solution of 10:1 wt/wt (≈5:1 mol/mol) BCF:F_4TCNQ in 95:5 vol/vol acetonitrile : ethyl acetate was coated as for BCF. Annealing and spin-off were performed identically to BCF.
- **F_4TCNQ:** 6 wt% solution in 95:5 vol/vol acetonitrile : ethyl acetate was blade-coated (40 °C; 6 mm s^{-1}) onto P3HT films, with all other details identical to doping with BCF.
- **$FeCl_3$:** 10 mM solution in acetonitrile was drop-cast onto the P3HT film to cover its entire area and held at room-temperature for 1 min, followed by removal by blow-drying. No spin-off was performed due to the known low stability of $FeCl_3$ doping.
- **LiTFSI:** 3.6 mg of $FeCl_3$ and 65.4 mg of LiTFSI were dissolved in 2.25 mL of acetonitrile. The solution was drop-cast onto the P3HT film to cover its entire area and held at room-temperature for 1 min, followed by removal by blow-drying. Excess dopant was removed by spin-off with 50 µL of acetonitrile at 3000 rpm.

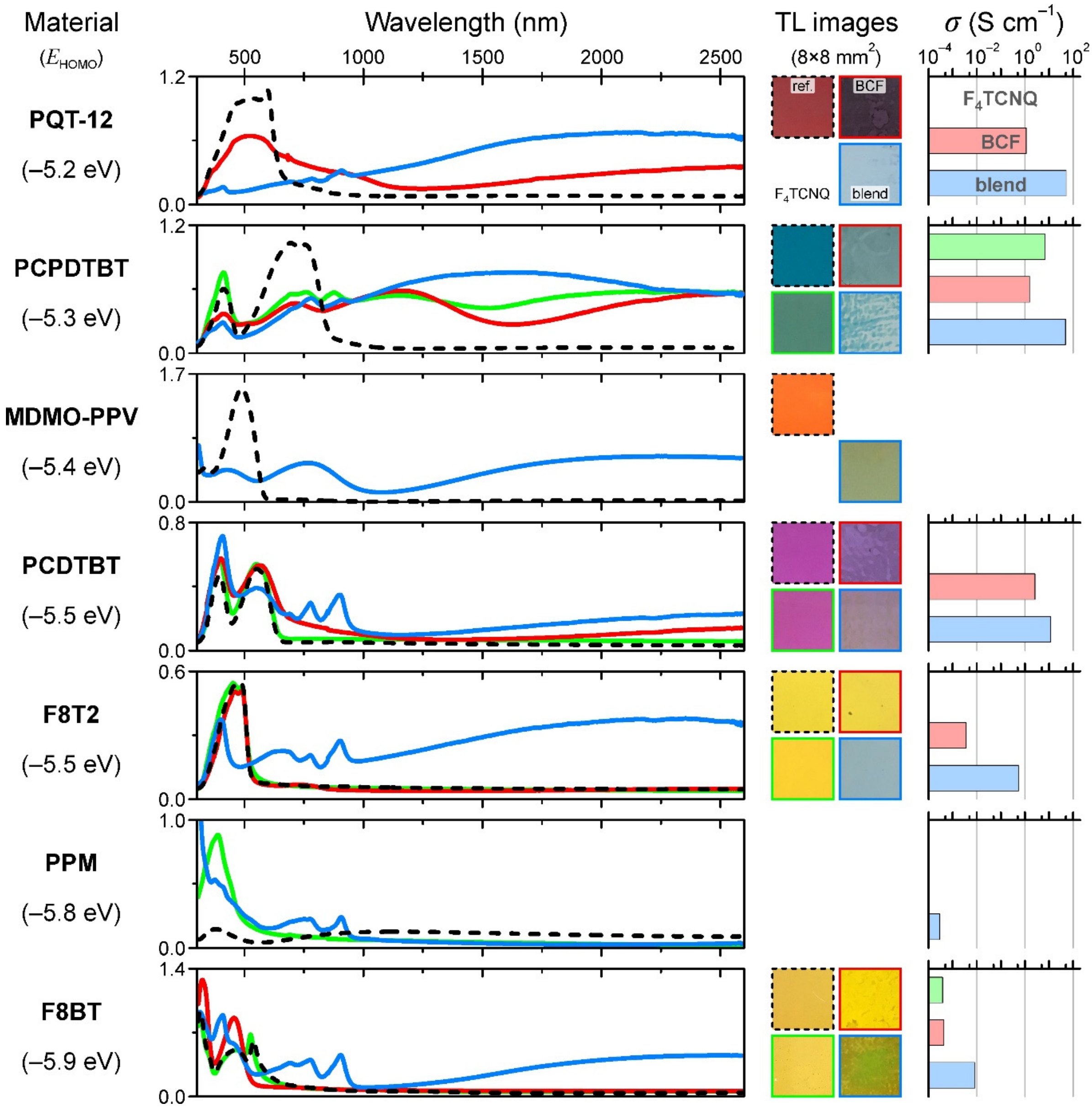

Figure S18. Summary of optical and electrical characteristics for a selection of macromolecular OSCs, arranged by increasing HOMO energies, showing UV-Vis-IR absorption spectra, transmitted-light (TL) images of ~8×8 mm^2 sample regions and electrical conductivities measured for the as-doped, unoptimized samples. Where available, data is shown for neat reference ('ref') thin films, and the same films doped with F_4TCNQ, BCF and 10:1 wt/wt BCF:F_4TNCQ blend.

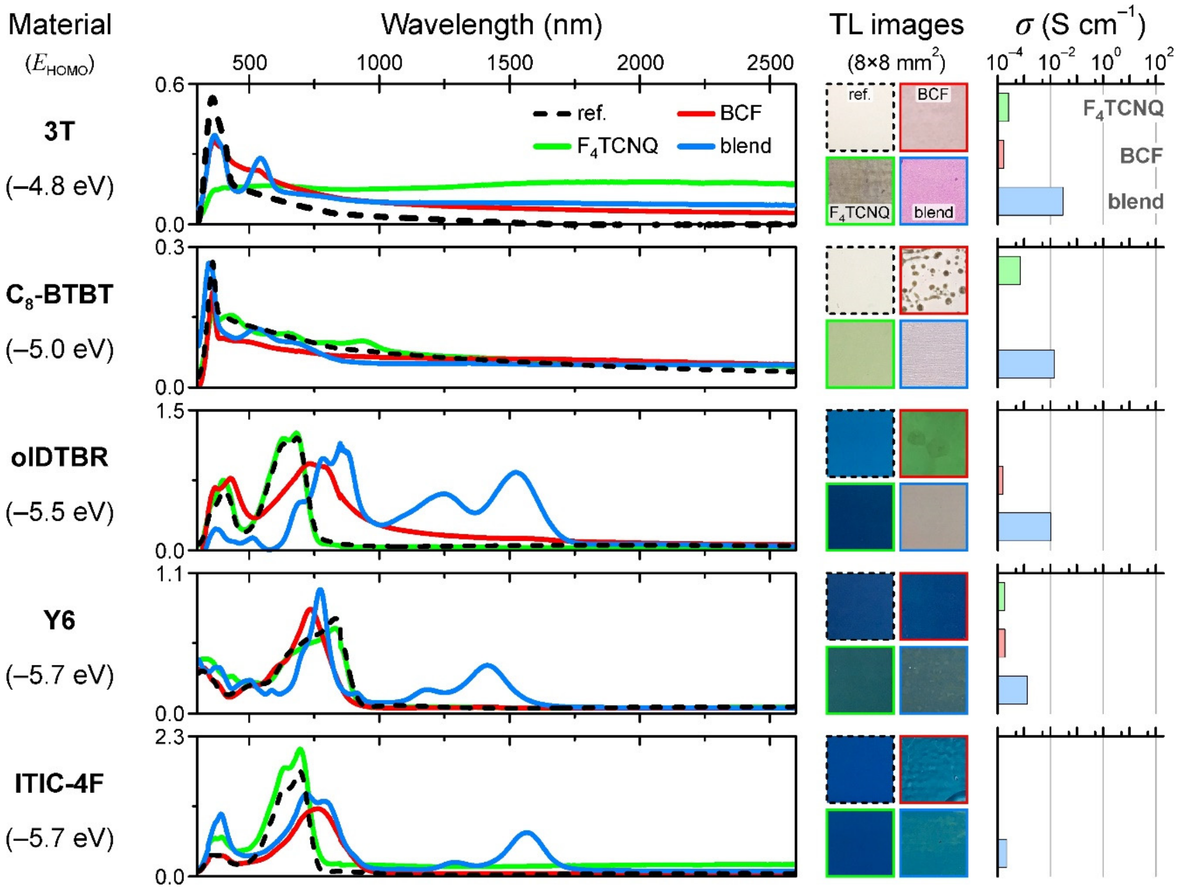

Figure S19. Summary of optical and electrical characteristics for a selection of benchmark small-molecular OSCs, arranged by increasing HOMO energies, showing UV-Vis-IR absorption spectra, transmitted-light (TL) images of ~8×8 mm^2 sample regions and electrical conductivities measured for the as-doped, unoptimized samples. In all cases, data is shown for neat reference ('ref') thin films, and the same films doped with F_4TCNQ, BCF and 10:1 wt/wt BCF:F_4TNCQ blend.

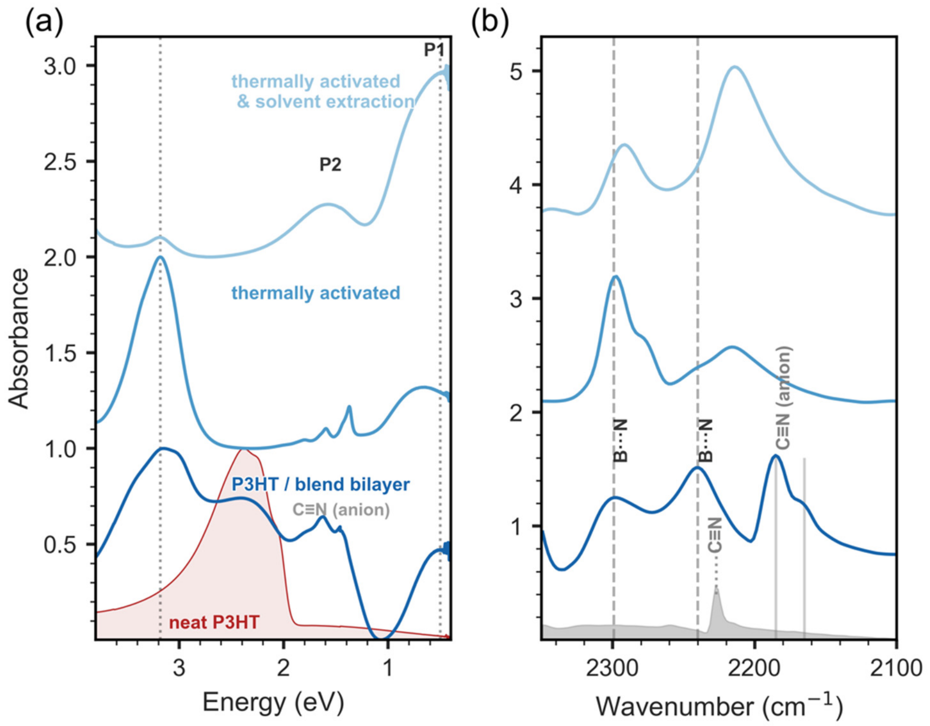

Figure S20. (**a**) UV-Vis-NIR and (**b**) IR spectra of neat P3HT and P3HT/BCF:F_4TCNQ bilayer before and after the thermal doping 'activation' process, as well as after subsequent solvent-based extraction. Dashed lines are a guide to the eye for representative features, i.e. the bands ascribed to F_4TCNQ or peaks ascribed to the neutral and anion forms of F_4TCNQ.

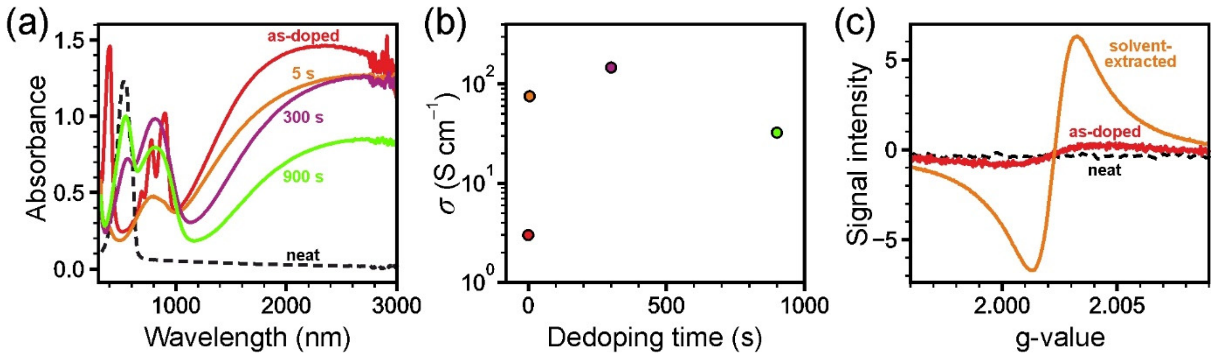

Figure S21. Optical and electronic properties of in-plane isotropic BCF:F_4TCNQ-doped P3HT films (thickness ≈ 140 nm): as-doped and following solvent-based extraction by immersion of doped films in acetone for the indicated durations. (**a**) UV-Vis-NIR spectra, (**b**) electrical conductivity and (**c**) EPR spectra.

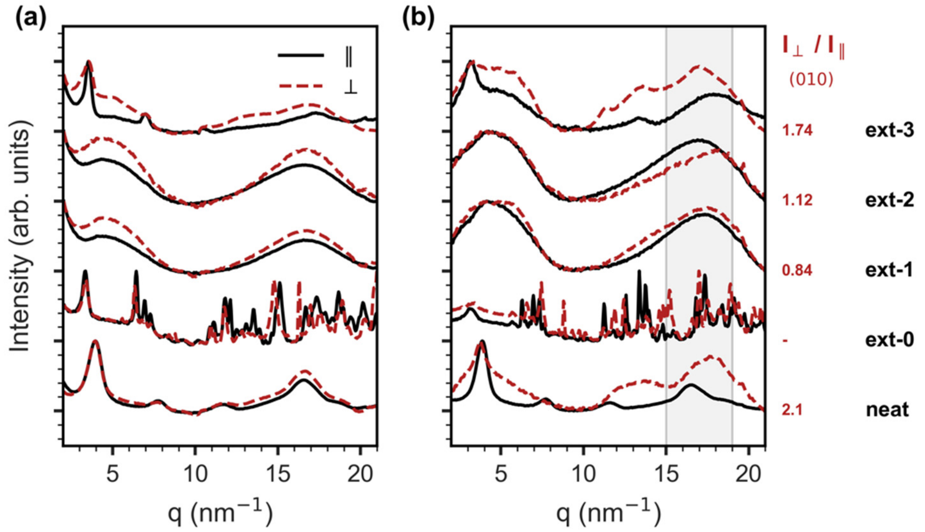

Figure S22. GIWAXS linecuts for the (**a**) out-of-plane and (**b**) in-plane directions for oriented films of P3HT. Measurements were taken with the X-ray beam oriented parallel and perpendicular to the chain orientation axis. Data shown neat P3HT and blend doped P3HT after incremental sequential solvent extraction steps. The shaded region highlights the peak used for calculating the dichroic ratio r_{I}.

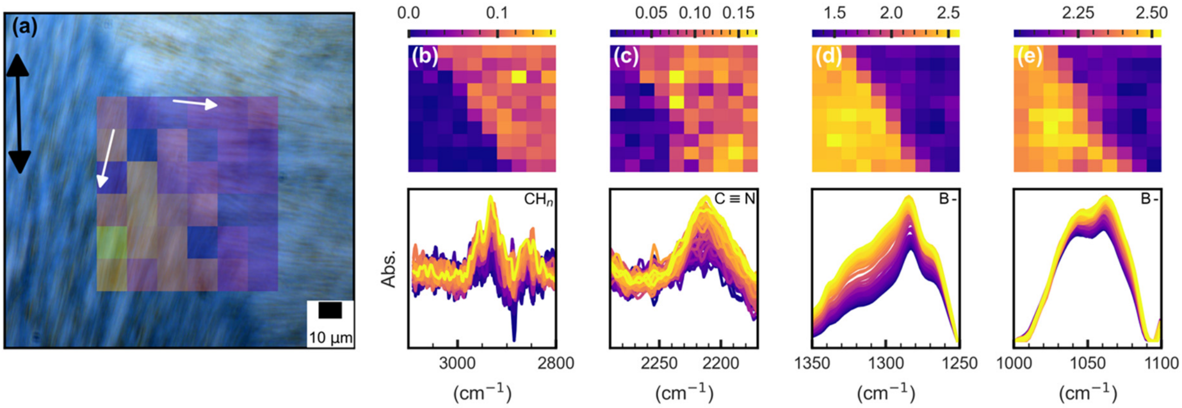

Figure S23. Data for BCF:F_4TCNQ-doped oriented P3HT, showing a microscopy image with an overlayed heatmap of the polarised IR map probing the polaron background from 1500 cm to 3700 cm^{-1}. Black arrow indicates the polarization direction and white arrows indicates the directions of local polymer chain alignment. Each pixel corresponds to a single measurement with a 10×10 μm^2 area. The colour scale indicates the area under the representative peaks. The additional included heatmaps probe other representative peaks shown below the corresponding panels.

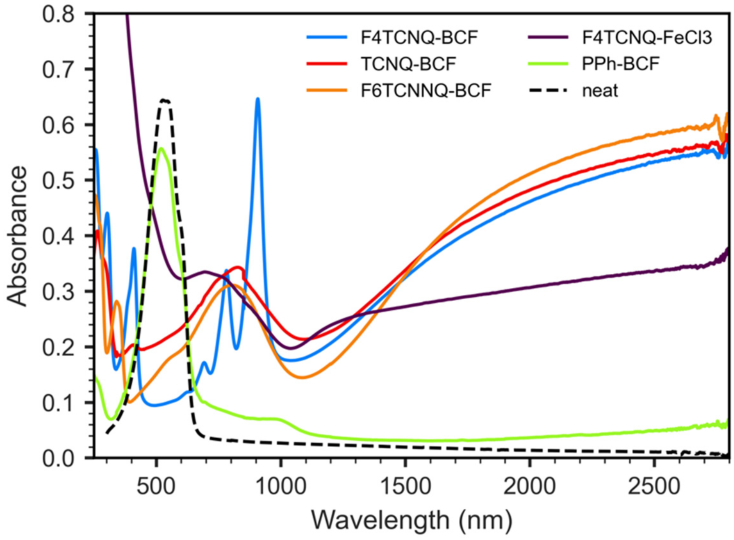

Figure S24. UV-Vis-NIR spectra of P3HT doped with different Lewis-paired complexes. All combinations, except for triphenyl phosphine (PPh) : BCF show doping capabilities.

3. Haacke figure-of-merit (φ_{TC}) for transparent conductive electrodes

Material/substrate	φ_{TC} at 550 nm ($10^{-3}\ \Omega^{-1}$)	Reference
ITO/glass	50	[8]
AgNW/PET	29	[9]
PEDOT:PSS/PET	9	[10]
PEDOT:PSS/glass	2	[11]
Graphene/glass	3	[12]
P3HT:BCF/glass	2×10^{-9}	This work; Fig. 1c
P3HT:F_4TCNQ/glass	2×10^{-8}	This work; Fig. 1c
P3HT:BCF:F_4TCNQ/glass	7×10^{-5}	This work; Fig. 1c
P3HT:BCF:F_4TCNQ/glass (*ext.*)	0.09	This work; Fig. 1c
PBTTT:BCF/glass	5×10^{-5}	This work; Fig. 4b
P3HT:F_4TCNQ/glass	7×10^{-3}	This work; Fig. 4b
P3HT:BCF:F_4TCNQ/glass	0.2	This work; Fig. 4b

Table S1. Haacke figure-of-merit values at 550 nm for a range of materials reported in the literature, as well as selected doped OSC films obtained in this work.

The most common figure-of-merit (FOM) employed for evaluating the performance of materials for transparent conductive electrodes (TCEs) is the Haacke FOM (φ_{TC}) defined as:[13]

$$\varphi_{\mathrm{TC}} = \frac{T^{10}}{R_s} = \sigma d \exp(-10\alpha d) \qquad (1)$$

where T is transmittance, R_s is sheet resistance, σ is electrical conductivity, d is film thickness and α is absorption coefficient. Typically, φ_{TC} is calculated from the corresponding T or α values at 550 nm.

Note that the φ_{TC} values for doped OSCs in Table 1 are likely to be underestimated since (i) the corresponding transmittance spectra were recorded without correction for specular reflection losses, as well as considering that (ii) the neutral polymer absorption for both P3HT and PBTTT is centered close to 550 nm.

4. References

1. Mejri, H.; Haidisch, A.; Krebsbach, P.; Seiberlich, M.; Hernandez-Sosa, G.; Perevedentsev, A. Gas-assisted blade-coating of organic semiconductors: Molecular assembly, device fabrication and complex thin-film structuring. *Nanoscale* **2022**, *14*, 17743–17753.
2. Perevedentsev, A.; Mejri, H.; Ruiz-Preciado, L. A.; Marszalek, T.; Lemmer, U.; Blom, P. W. M.; Hernandez-Sosa, G. Polarization-sensitive photodetectors based on directionally oriented organic bulk-heterojunctions. *Adv. Optical Mater*. **2022**, *10*, 2102397.
3. Kresse, G.; Furthmüller, J. Efficient iterative schemes for ab initio total-energy calculations using a plane-wave basis set. *Phys. Rev. B* **1996**, *54*, 11169–11186.
4. Blöchl, P. E. Projector augmented-wave method. *Phys. Rev. B* **1994**, *50*, 17953–17979.
5. Kresse, G.; Joubert, D. From ultrasoft pseudopotentials to the projector augmented-wave method. *Phys. Rev. B* **1999**, *59*, 1758–1775.
6. Perdew, J. P.; Burke, K.; Ernzehof, M. Generalized gradient approximation made simple. *Phys. Rev. Lett*. **1996**, *77*, 3865–3868.
7. Dörling, B.; Rodríguez-Martínez, X.; Álvarez-Corzo, I.; Reparaz, J. S.; Campoy-Quiles, M. Soluble alkali-metal carbon nanotube salts for n-type thermoelectric composites with improved stability. *Appl. Phys. Lett*. **2021**, *118*, 213901.
8. Fraser, D. B.; Cook, H. D. Highly conductive, transparent films of sputtered In_{2-x} Sn_x O_{3-y}. *J. Electrochem. Soc*. **1972**, *119*, 1368–1374.
9. Andrés, L. J.; Menéndez, M. F.; Gómez, D.; Martínez, A. L.; Bristow, N.; Kettle, J. P.; Menéndez, A.; Ruiz, B. Rapid synthesis of ultra-long silver nanowires for tailor-made transparent conductive electrodes: Proof of concept in organic solar cells. *Nanotechnology* **2015**, *26*, 265201.
10. Hu, X.; Meng, X.; Zhang, L.; Zhang, Y.; Cai, Z.; Huang, Z.; Su, M.; Wang, Y; Li, M.; Li, F.; Yao, X.; Wang, F.; Ma, W.; Chen, Y.; Song, Y. A Mechanically robust conducting polymer network electrode for efficient flexible perovskite solar cells. *Joule* **2019**, *3*, 2205–2218.
11. Jeong, H. J., Jang, H., Kim, T., Earmme, T. & Kim, F. S. Sigmoidal dependence of electrical conductivity of thin PEDOT:PSS films on concentration of linear glycols as a processing additive. *Materials* **2021**, *14*, 1975.
12. Mirri, F.; Ma, A. W. K.; Hsu, T. T.; Behabtu, N.; Eichmann, S. L.; Young, C. C.; Tsentalovich, D. E.; Pasquali, M. High-performance carbon nanotube transparent conductive films by scalable dip coating. *ACS Nano* **2012**, *6*, 9737–9744.
13. Haacke, G. New figure of merit for transparent conductors. *J. Appl. Phys*. **1976**, *47*, 4086–4089.
